# Favorable Desolvation and Uniform Zn Deposition of Silica Modified Zn Anode for High Performance Aqueous Zn‐Ion Batteries

**DOI:** 10.1002/advs.202417121

**Published:** 2025-04-02

**Authors:** Wangsheng Yuan, Kai Jin, Shanbao Zou, Yishan Jin, Yishuang He, Xinhai Yuan, Peng Han, Lijun Fu, Yuping Wu

**Affiliations:** ^1^ State Key Laboratory of Materials‐Oriented Chemical Engineering School of Chemistry and Molecular Engineering and College of Energy Science and Engineering Nanjing Tech University Nanjing 211816 P. R. China; ^2^ Department of Physics Capital Normal University Beijing 100048 P. R. China

**Keywords:** aqueous zinc‐ion batteries, desolvation, fumed silica, homogeneous Zn deposition, zinc anode coating

## Abstract

The instability of Zn anode resulting from corrosion and dendritic growth remain impeding the development of aqueous zinc‐ion batteries (AZIBs). In addition, the desolvation on the Zn surface is sluggish, which hinders the reaction kinetics and fast charge/discharge behavior of AZIBs. Herein, the uniform Zn deposition and fast desolvation are realized by using a hydrophilic fumed nano‐silica coating with zinc alginate (Alg) as a functional binder (Alg/SiO_2_@Zn). Combined theoretical calculation and experimental investigations show the interaction between the H_2_O in the solvation structure and ‐OH functional group of SiO_2_ facilitates the desolvation process. In addition to the Zn^2+^ guiding effect of Alg, the fast Zn^2+^ diffusion along SiO_2_ endows the homogeneous Zn deposition. Consequently, Alg/SiO_2_@Zn symmetric cell demonstrates exceptional plating/stripping reversibility and excellent long‐term cycle life at both 1 and 10 mAh cm^−2^. A full cell assembled with Alg/SiO_2_@Zn and NaV_3_O_8_·*x*H_2_O cathode achieves a high capacity of 129 mAh g^−1^ at 4 A g^−1^ over 2650 cycles. Even under −20 °C, the battery maintains a high capacity of 136.5 mAh g^−1^ at 1 A g^−1^ after 1000 stable cycles. This study provides a facile strategy to achieve a highly stable Zn anode and gains deep insight into desolvation modulation via surface modification.

## Graphical abstract

The uniform Zn deposition and fast desolvation of Zn anode are realized by using a hydrophilic fumed nano‐silica coating with zinc alginate (Alg) as a functional binder. The interaction between the H_2_O in the solvation structure and ‐OH functional group of SiO_2_ facilitates the desolvation process. In addition to the Zn^2+^ guiding effect of Alg, the fast Zn^2+^ diffusion along SiO_2_ endows the homogeneous Zn deposition.

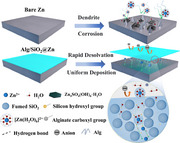

## 1 Introduction

Aqueous zinc‐ion batteries (AZIBs) hold promising application potential for large‐scale energy storage, since they present high safety, low cost, environmental friendliness, and high ionic conductivity.^[^ ^1^ ^]^ However, the Zn dendrite growth resulting from the ongoing plating/stripping cycle of zinc metal is detrimental to the cycle stability.^[^ ^2^ ^]^ Moreover, due to thermodynamic factors, zinc metal tends to induce substantial hydrogen evolution reaction in water, leading to side reactions and subsequent poor electrochemical performance.^[^ ^3^ ^]^ These factors significantly impede their commercial viability for large‐scale energy storage applications.^[^ ^4^ ^]^

Current research focused on optimizing zinc anodes encompasses the structural design of zinc metal anodes,^[^ ^5^ ^]^ electrolyte conditioning,^[^ ^3^, ^6^ ^]^ and anode/electrolyte interface modification.^[^ ^7^ ^]^ As dendrites and side reactions primarily occur at the zinc anode/electrolyte interface, the introduction of artificial interface layers is deemed the most straightforward and efficacious strategy.^[^ ^8^ ^]^ To date, the application of inorganic coatings as interfacial modifiers for zinc anodes has been reported in numerous studies, primarily involving metal oxides (ZnO,^[^ ^9^ ^]^ Al_2_O_3_,^[^ ^10^ ^]^ TiO_2_,^[^ ^11^ ^]^ ZrO_2_,^[^ ^12^ ^]^ SiO_2_^[^ ^13^ ^]^), fluorides (ZnF_2_^[^ ^14^ ^]^), and phosphates (Zn(H_2_PO_4_)_2_^[^ ^15^ ^]^). These materials significantly enhance the performance of AZIBs by forming a stable protective layer on the surface of the zinc anode. However, under prolonged cycling or extreme conditions, rigid inorganic coatings may undergo physical or chemical changes, such as cracking, delamination. Gel polymer coatings are proved to play an effective role in inhibiting zinc anode dendrites, meanwhile alleviating the problems resulted from the rigid properties of inorganic coating. Previously we reported for the first time on zinc alginate (Alg) as a zinc anode coating and elucidated the mechanism of how the zinc ions were guided in the coating and thus the Zn dendrite formation was suppressed.^[^ ^16^ ^]^ In addition, the acrylamide (AAm) coating^[^ ^17^ ^]^ and polyacrylonitrile (PAN) coating^[^ ^18^ ^]^ could direct the homogeneous deposition of zinc ions. Although these polymers with polar functional groups strongly interact with zinc ions, facilitating a more compact and ordered zinc ion deposition, this strong interaction also limits the transport rate of zinc ions, resulting in a significantly increased polarization.^[^ ^19^ ^]^

The desolvation of hydrated zinc ions is identified as a crucial factor constraining anode deposition kinetics.^[^ ^20^ ^]^ Elevated desolvation energy hampers the swift and even zinc ion deposition on the zinc anode, and the active water molecules released during desolvation are prone to corroding the zinc surface, exacerbating undesirable side reactions. Therefore, by adjusting the solvation structure of hydrated zinc ions, the side reactions of electrode materials can be effectively inhibited, and the cyclic stability and electrochemical kinetic performance of the battery can be improved. Electrolyte component adjustment and coordinated interface design were commonly used to adjust the Zn^2+^ solvation structure. For the former case, functional additives or cosolvents were added. For the latter case, gel‐polymer or nanometallic organic frame (MOF) material coating was introduced on the surface of Zn, which could coordinate Zn^2+^ and regulate the solvation structure. However, the complex electrolyte composition may adversely affect the positive electrode or the battery's rate performance.^[^ ^19^ ^]^ Meanwhile, the gel polymer results in low ionic conductivity and high overpotential and the MOF materials would increase the manufacturing cost.^[^ ^19^ ^]^ Therefore, it is of great significance to explore simple, safe, and low‐cost strategy for zinc ion solvation structure regulation.

Silicon dioxide, known for its high chemical stability and low cost, has been investigated as an anode coating for AZIBs. When SiO_2_ was combined with PVDF to form an artificial interfacial coating for zinc anodes, SiO_2_ acted as an ion sieve and could effectively suppress the formation of dendrites at the zinc anode, allowing for stable cycling of Zn anode for 1000 h at a current density of 1 mA cm^−2^.^[^ ^13^ ^]^ Composite of zinc alginate and SiO_2_ as an artificial interfacial coating for zinc anodes was reported, the Zn^2+^ redistribution effect of SiO_2_ at the interface is described by simulating the concentration field of zinc ion at the interface.^[^ ^21^ ^]^ These reports demonstrated the efficacy of SiO_2_ dioxide in inhibiting dendritic growth on zinc anodes. Our previous report has also shown that alumina coating has a good effect on inhibiting dendrites.^[^ ^22^ ^]^ In addition, with those coating, the rate performance of the modified Zn anode and the corresponding AZIBs was enhanced. It should be noted that the reported coating layer contains polymers such as PVDF or zinc alginate, which interact strongly with the Zn^2+^ and lead to high polarization and poor performance at high current density. Thus, the silica is pivotal in accelerating the reaction kinetics of Zn stripping and plating and it is significant to explore the function underneath.

Herein, hydrophilic fumed silica is used as the modification coating for Zn anode with Alg or PVDF as binders, the prepared zinc anodes are labeled as Alg/SiO_2_@Zn and PVDF/SiO_2_@Zn, respectively. Both the Alg/SiO_2_@Zn and PVDF/SiO_2_@Zn show improved reaction kinetics and cycle stability, in comparison to the bare Zn. The SiO_2_ content in the coating enhances the Young's modulus and provides rich array of negative charges, which effectively regulate the preferential homogenized deposition of zinc ions along Zn (002) and inhibit the side reaction during the cycling process. Combined experimental and theoretical calculations show that SiO_2_ can effectively promote the desolvation process of hydrated zinc ions at the interface, which is due to the fixation of water molecules in the solvated sheath by SiO_2_‐OH. In addition, SiO_2_ significantly reduces the polarization and enhances the deposition/stripping kinetics. The Alg/SiO_2_@Zn symmetric cell demonstrates remarkable stability and rate performance, ≈3000 h cycling at a current density of 1 mA cm^−2^ and an area capacity of 1 mAh cm^−2^ can be achieved. Even under a high current density of 10 mA cm^−2^ and an area capacity of 10 mAh cm^−2^, it can still cycle stably for 340 h. Moreover, when combined with an activated carbon (AC)/sodium vanadate (NVO) cathode, the Alg/SiO_2_@Zn anode showcases exceptional cycling stability. It also demonstrates excellent electrochemical performance even in frozen conditions at −20 °C. These findings provide a solid foundation for the future development of dendrite‐free, long‐life, and high‐rate AZIBs, and open up possibilities for their broader application.

## 2 Results and Discussion

In this work, sodium alginate and hydrophilic fumed nano‐SiO_2_ were thoroughly mixed in deionized water. The resulting gelled mixture was then uniformly applied to the surface of zinc foil using a straightforward drop coating method. When Na^+^ was replaced by Zn^2+^ in the electrolyte, an Alg/SiO_2_ coating capable of transferring zinc ions could be in situ formed, the modified anode is denoted as Alg/SiO_2_@Zn (Figure S1, Supporting Information). For comparison, a control sample of zinc alginate coating (Alg@Zn) was prepared without the addition of SiO_2_.

**Figure 1:**
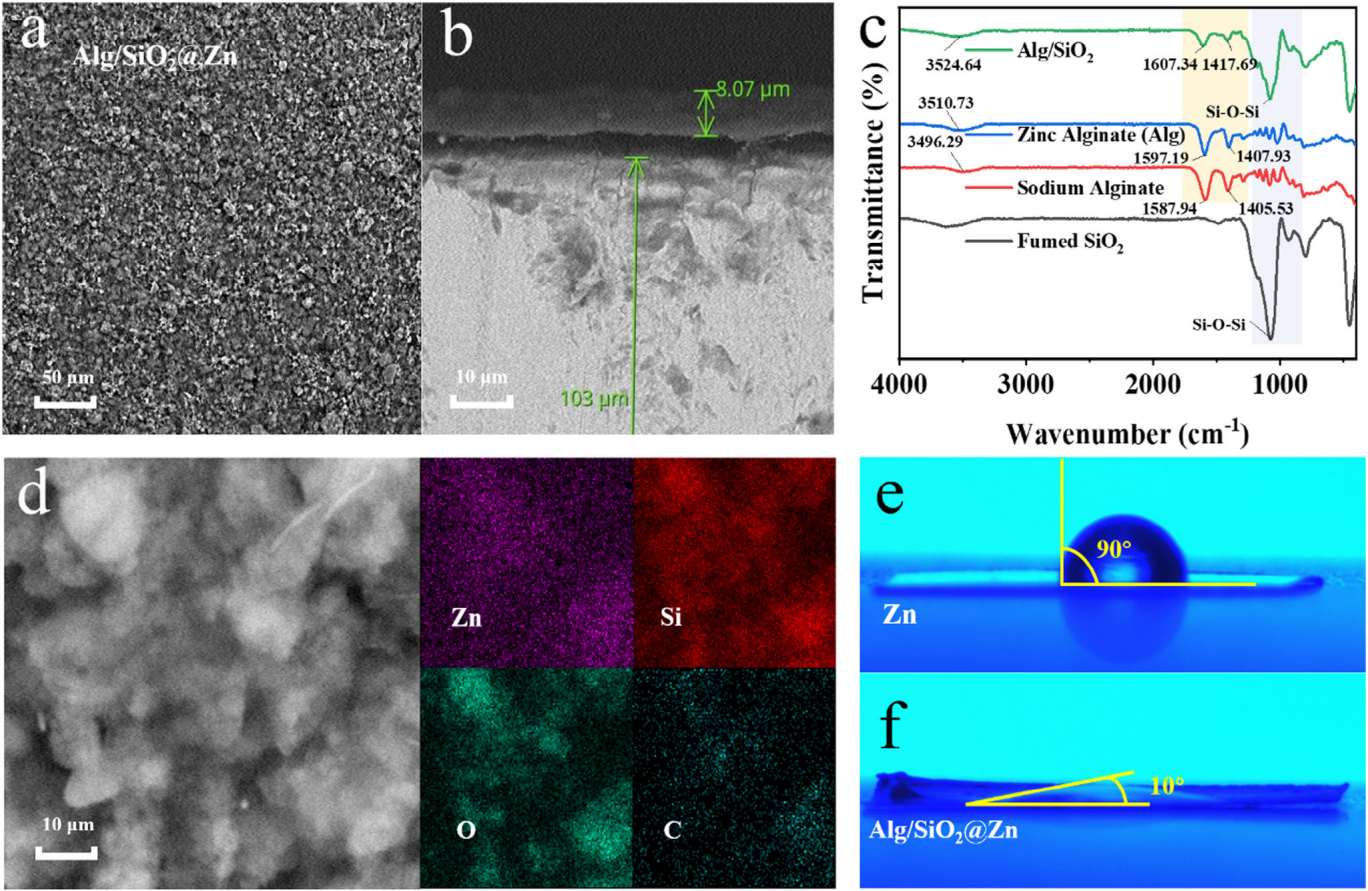
Physical characterization of different zinc anodes. a) planar and b) section SEM of Alg/SiO_2_@Zn. c) FTIR of sodium alginate, Alg, fumed SiO_2_, and Alg/SiO_2_. d) EDS of the initial Alg/SiO_2_@Zn plane. Contact angle measurements for e) bare Zn and f) Alg/SiO_2_@Zn.

The crystal structures and morphologies of bare Zn, Alg@Zn, and Alg/SiO_2_@Zn were first characterized by scanning electron microscopy (SEM), respectively. Polishing marks could be clearly observed on the bare Zn anode surface (Figure S2a, Supporting Information), and for Alg@Zn, a flat Zn alginate hydrogel coating is observed (Figure S2b, Supporting Information). In contrast, the Alg/SiO_2_@Zn surface exhibits a homogeneous granular distribution (**Figure** **1**a), because of the presence of SiO_2_, which exhibits a large specific surface area. Furthermore, the thickness of the Alg/SiO_2_ coating (≈8 µm) is well suited for the kinetics of zinc ion diffusion (Figure 1b). The X‐ray diffraction (XRD) patterns of the two modified zinc foils exhibit diffraction peaks that align with those of the bare Zn (Figure S3, Supporting Information). These diffraction peaks correspond to the standard PDF card (JCPDS 04–0831), indicating that the crystal structure of the modified foils remains as that of the unmodified bare Zn. The hydrophilic fumed nano‐SiO_2_ possesses an amorphous structure (Figures S4 and S5, Supporting Information). The presence of Alg and SiO_2_ in the coating can be confirmed through Fourier transform infrared spectroscopy (FTIR) characterization (Figure 1c). For the nano‐SiO_2_, the peak exists at 1077 cm^−1^ is attributed to Si‐O‐Si bond. For the sodium alginate, the peak observed at 3496.29 cm^−1^ corresponds to the O‐H stretching vibration of hydrogen bonds within the alginate polymer chain. The peaks at 1587.94 cm^−1^ and 1405.53 cm^−1^ are attributed to the asymmetric and symmetric stretching vibrations of the ‐COO‐ group in the alginate chain respectively. The spectrum of zinc alginate is similar to that of sodium alginate, while the peaks of the ‐COO‐ group are blue shifted from 1587.94 and 1405.53 cm^−1^ to 1597.19 and 1407.93 cm^−1^, which is probably due to the coordination between the carboxylate group and the zinc ion.^[^ ^16^ ^]^ This change arises from alterations in both the charge density and radius of the cation upon substitution of the Na^+^ with the Zn^2+^, consequently leading to the formation of a new environment surrounding the carbonyl group.^[^ ^23^ ^]^ As to the Alg/SiO_2_, the peaks of the ‐COO‐ group are further blue shifted to 1607.34 cm^−1^ and 1417.69 cm^−1^, which may be due to the influence of functional groups in SiO_2_. The uniform distribution of the element Si in the coating was clearly observed by Energy dispersive spectroscopy (EDS) investigation of Alg/SiO_2_@Zn (Figure 1d; Figure S6, Supporting Information). The results confirm that the Alg/SiO_2_ coating is a homogeneous composite of zinc alginate and silicon dioxide and is flatly encapsulated on the surface of the zinc foils.

The wettability of the electrode plays a crucial role in influencing the kinetics of the electrode reaction. In order to evaluate the wettability of the 2 M ZnSO_4_ electrolyte on bare Zn and two modified coatings, contact angle measurements were conducted. The contact angles for bare Zn, Alg@Zn, and Alg/SiO_2_@Zn are determined to be 90°, 20°, and 10°, respectively (Figure 1e,f; Figure S7, Supporting Information). It can be observed that both Alg@Zn and Alg/SiO_2_@Zn exhibit smaller contact angles, indicating improved wettability compared to bare Zn after surface coating modification. Particularly, Alg/SiO_2_@Zn displays the smallest contact angle, which can be attributed to the increased specific surface area and the high hydrophilicity of SiO_2_ and Alg. Enhanced wettability promotes the efficient transfer of zinc ions across the electrode/electrolyte interface, thereby improving the kinetics of zinc ion plating/stripping. Furthermore, bare Zn and zinc foils covered with different coatings were immersed in the 2 M ZnSO_4_ electrolyte for 7 days, the results show that the Alg/SiO_2_@Zn coating exhibits better self‐corrosion inhabitation ability than Alg@Zn and bare Zn, providing reliable protection for the Zn anode (Figures S8‐S10, Supporting Information). In addition, the mechanical strength of Alg and Alg/SiO_2_ coatings is evaluated via stress‐strain tests. The results indicate that both the Alg and Alg/SiO_2_ hydrogel films demonstrate outstanding tensile strains, with values of 62% and 53%, respectively. Moreover, the films exhibit high Young's modulus values, 0.22 MPa for Alg and 0.25 MPa for Alg/SiO_2_ (Figures S11 and S12, Supporting Information). It can be found that the incorporation of SiO_2_ into the Alg hydrogel film leads to a slight decrease in flexibility but an increase in mechanical strength compared to the Alg hydrogel film, which would not only facilitate the accommodation of volume change during the deposition/plating process, but also serves as a physical shelter against dendrite growth formation.

**Figure 2:**
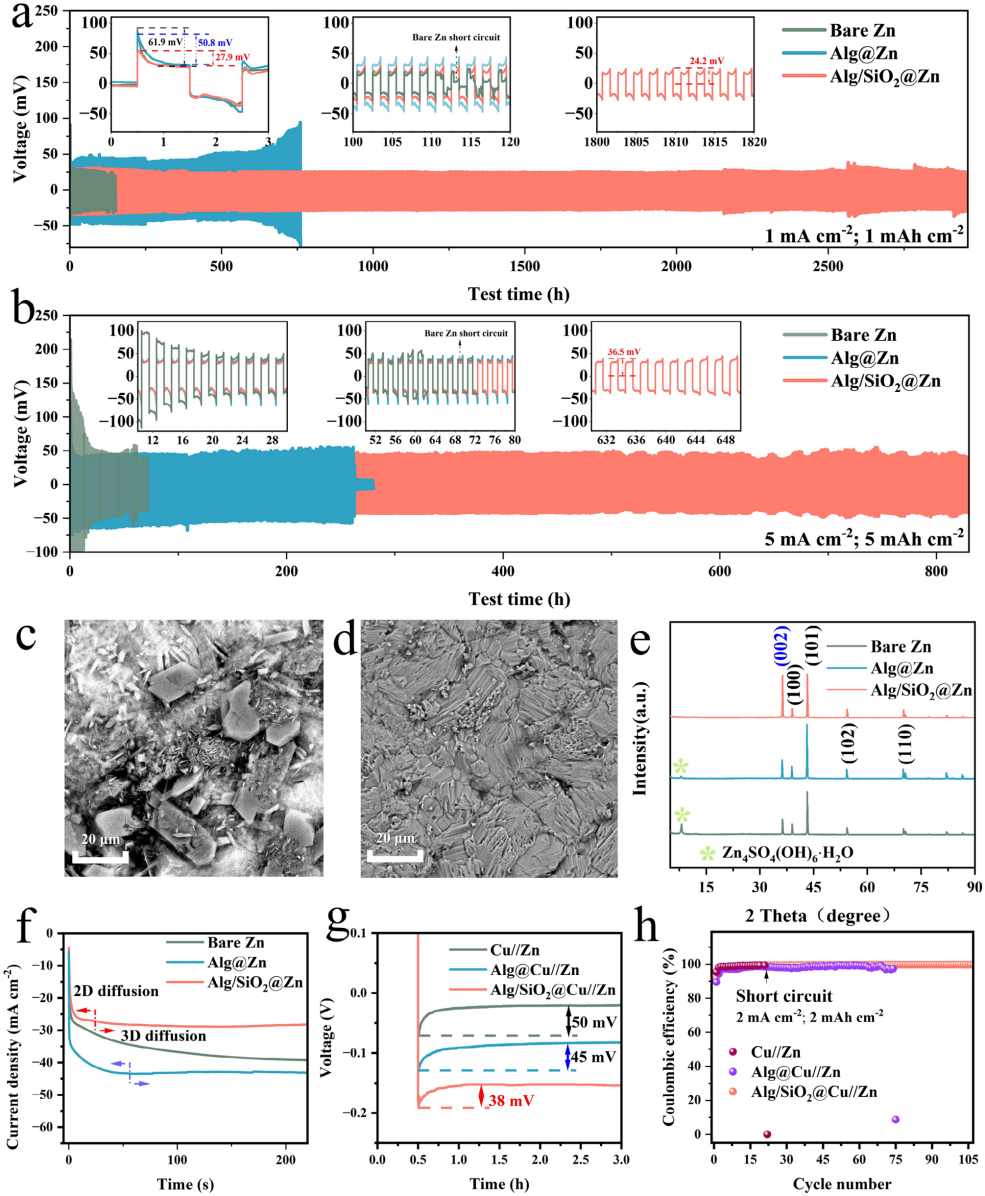
Electrochemical performance of symmetrical Zn cells with bare Zn, Alg@Zn, and Alg/SiO_2_@Zn electrodes. a) Galvanostatic charge‐discharge profiles at a current density of 1 mA cm^−2^ and areal capacity of 1 mAh cm^−2^, the initial zinc nucleation and growth overpotential profiles are shown in the small figure. b) Galvanostatic charge‐discharge profiles at a current density of 5 mA cm^−2^ and an areal capacity of 5 mAh cm^−2^. SEM of c) bare Zn and d) Zn foil with Alg/SiO_2_ coating removed after 50 cycles (100 h). e) XRD of the deposited side of bare Zn, Alg@Zn, and Alg/SiO_2_@Zn after 50 cycles (100 h). f) The chronoamperometry (CA) curves of bare Zn and Alg/SiO_2_@Zn at a ‐150 mV overpotential. g) Nucleation growth overpotential and h) the Coulombic efficiency of Cu//Zn, Alg@Cu///Zn, and Alg/SiO_2_@Cu//Zn asymmetric cells.

Symmetric cells with bare Zn, Alg@Zn, and Alg/SiO_2_@Zn were assembled for electrochemical evaluation. At a current density of 1 mA cm^−2^ and a surface capacity of 1 mAh cm^−2^ (**Figure** **2**a), cells with bare Zn and Alg@Zn electrodes demonstrate a higher nucleation growth overpotential of 61.9 and 50.8 mV, respectively. In comparison, Alg/SiO_2_@Zn exhibits a significantly lower nucleation growth overpotential of 27.9 mV. During the subsequent plating/stripping, bare Zn exhibits a polarization potential of 25.1 mV (the difference between the plating/stripping tip potential and 0 V). The smaller polarization of bare Zn afterwards might be attributed to the dendrite formation during cycling. As cycling progresses, bare Zn experiences the dendrite formation on the surface. These dendrites increase the reactive surface area, leading to faster deposition/stripping kinetics and lower polarization. The polarization potential of Alg@Zn is 43.8 mV. The increased polarization observed in Alg@Zn can be attributed to the strong binding energy between the carboxyl group in Alg and Zn^2+^, which hinders the migration of Zn^2+^ within the coating.^[^ ^16^ ^]^ For Alg/SiO_2_@Zn, the polarization potential is 27.9 mV, which is only slightly higher than that of bare Zn, but much lower than that of Alg@Zn. After cycling for 1800 h, the polarization potential of Alg/SiO_2_@Zn is further reduced to 24.2 mV. The results suggest that with the addition of SiO_2_, the strong interaction between Alg and Zn^2+^ is effectively reduced, leading to a notable reduction of polarization and favorable reaction kinetics of Alg/SiO_2_@Zn, in comparison to Alg@Zn. For bare Zn, after 112 h of cycling, it exhibits a significant voltage profile fluctuation followed by a short circuit, resulting from the dendrite growth.^[^ ^24^ ^]^ In contrast, Alg/SiO_2_@Zn demonstrates an impressive cycle life of ≈3000 h, nearly 30 times longer than bare Zn and outperforming Alg@Zn (≈700 h) as well. At a current density of 5 mA cm^−2^ and an area capacity of 5 mAh cm^−2^ (Figure 2b), bare Zn experiences a significant increase in polarization in comparison to that at 1 mA cm^−2^ and 1 mAh cm^−2^, with a polarization potential of 51.2 mV, which ultimately leads to a short‐circuit after 60 h of cycling. On the other hand, Alg@Zn and Alg/SiO_2_@Zn demonstrate polarization potential of 54.3 mV and 38.8 mV, respectively. In contrast to the short cycle life of bare Zn metal, Alg@Zn, and Alg/SiO_2_@Zn achieve an extended cycling of 265 h and 860 h, respectively. When the current density is increased to 10 mA cm^−2^ and the area capacity is set as 10 mAh cm^−2^ (Figure S13, Supporting Information), bare Zn can barely be cycled, and Alg@Zn presents a short cycle life of 130 h, while Alg/SiO_2_@Zn demonstrates excellent cycle stability of ≈380 h. The results suggest that at high current density (such as 10 mA cm^−2^), SiO_2_ has a very significant effect on promoting the reaction kinetics. The rate performance of the symmetrical cells was tested with an area capacity of 1 mAh cm^−2^ and different current densities (Figure S14, Supporting Information). At high current densities of 10 mA cm^−2^, Alg/SiO_2_@Zn exhibits the smallest polarization. This also proves that SiO_2_ coating can optimize the kinetics of zinc anode. Notably, the electrochemical performance of the Alg/SiO_2_@Zn symmetric cell is superior to the previously reported artificial SEI‐modified zinc anodes, as shown in Table S1 (Supporting Information).

To ensure the important role of SiO_2_ in the coating layer in enhancing the rate performance of Zn electrode, PVDF was selected as an alternative binder, and PVDF/SiO_2_ modified Zn electrode (PVDF/SiO_2_@Zn) was prepared, with a weight ratio of 1:1. In symmetric cell testing with a current density of 1 mA cm^−2^ and an area capacity of 1 mAh cm^−2^, the PVDF/SiO_2_@Zn exhibits a lower polarization of 20 mV compared to the polarization observed for bare Zn (25.1 mV). Furthermore, the PVDF/SiO_2_@Zn demonstrated stable cycling for ≈1330 h, indicating improved cycling performance (Figure S15, Supporting Information). The rate performance of the symmetric cell is also enhanced with the PVDF/SiO_2_@Zn, the polarization potential is only 55 mV at a current density of 10 mA cm^−2^ (Figure S16, Supporting Information), whereas PVDF@Zn is unable to cycle properly at the same current density (Figure S17, Supporting Information). It is worth noting that due to the strong hydrophobicity of PVDF, it may often lead to worse rate performance. The PVDF/SiO_2_@Zn anode, on the other hand, shows excellent rate performance, even better than the Alg/SiO_2_@Zn anode. We consider that the occurrence of this situation may be due to the strong interaction between Alg and zinc ions, which to a certain extent counteracts the positive effect of SiO_2_ coating on the deposition/stripping kinetics of zinc ions. All in all, it is evidenced that SiO_2_ coating can optimize the reaction kinetics of zinc anode.

Surface analyses of zinc foils in the symmetric cells were carried out. After 10 h of unidirectional deposition at a current density of 1 mA cm^−2^, unevenly distributed flakes and mossy deposits are observed on the surface of bare Zn (Figure S18a, b, Supporting Information). For Alg@Zn, no flake‐like deposition is observed when removing the Alg coating, but uneven deposition is still evident (Figure S18c, d, Supporting Information). In contrast, the Alg/SiO_2_@Zn presents homogeneous and dense deposition on the Zn metal surface, when the Alg/SiO_2_ coating is removed (Figure S18e, f, Supporting Information). When the symmetric cells were cycled for 50 times at a current density of 1 mA cm^−2^ and an area capacity of 1 mAh cm^−2^, the bare Zn surface become quite rough, with large flaky byproducts and dendrites on the surface (Figure 2c). For Alg@Zn electrode, upon removing the Alg coating, though no large flaky by‐products are observed on the modified zinc foil surface, small dendrite‐like zinc aggregates can still be observed after several cycles, which are not conducive to long‐term cycling (Figure S19, Supporting Information).^[^ ^16^ ^]^ In contrast, the morphology of Alg/SiO_2_@Zn after cycling remains similar to its initial state, exhibiting excellent structural stability (Figure S20, Supporting Information). This observation suggests that the Alg/SiO_2_ coating maintains its integrity and retains its original structural properties even after repeated cycling. Upon removing the Alg/SiO_2_ coating, the surface of the modified zinc foil exhibits a more uniform and flat morphology after cycling (Figure 2d). EDS testing of the Alg/SiO_2_@Zn after 50 cycles at deposited state reveals that the atomic ratio of Si: Zn elements in the coatings remains at 2.8 (Figure S21 and Table S2, Supporting Information), which is similar to the atomic ratio of Si: Zn elements on the initial coating surface (≈2.8). This indicates that Zn ions are able to pass through the coatings and deposit uniformly on the Zn metal surface. These findings align with the previous reports, which showed that the carboxylic acid groups in Alg interacted with Zn^2+^ and directed the diffusion of Zn^2+^ via electrostatic forces, eventually the Zn was deposited on the zinc metal under the Alg coating.^[^ ^16^, ^23^ ^]^ XRD tests reveal that no obvious trace of by‐products Zn_4_SO_4_(OH)_6_·H_2_O is observed at the surface of Alg/SiO_2_ coating modified zinc foil, while the by‐products are formed on the bare Zn and Alg coating modified zinc foil (Figure 2e). Zeta potential of SiO_2_ in deionized water was measured as ‐38.8 mV (Figure S22, Supporting Information) and the negative charges present on SiO_2_ exhibit an electrostatic shielding effect on the SO_4_^2−^ anions, which would benefit the inhibition of Zn_4_SO_4_(OH)_6_·H_2_O by‐product formation. Interestingly, we find that the peak intensity on the (002) crystal plane of the deposited zinc is significantly enhanced for the Alg/SiO_2_@Zn after 50 cycles. For the bare Zn and Alg@Zn, (101) crystal plane is the preferential growth orientation. The XRD results suggest that SiO_2_ is able to induce the preferential deposition of zinc ions on the (002) crystal plane, which is conducive to the achievement of a dendrite‐free zinc anode, at the same time, the side reaction is inhibited by the SiO_2_ containing coating layer. In situ optical microscopy observation shows that Alg/SiO_2_@Zn exhibits a flat surface during the whole 30 min deposition process, while plenty of inhomogeneous deposits are observed for the bare Zn and Alg@Zn, further suggesting the Alg/SiO_2_ coating is efficient in guiding the uniform Zn deposition (Figure S23, Supporting Information).

The nucleation and growth behavior of Zn metal was studied by chronoamperometry (CA) (Figure 2f).^[^ ^25^ ^]^ For the bare Zn electrode, the current density continuously increases for over 180 s when a polarization of ‐150 mV is applied. This behavior indicates the 2D diffusion of the adsorbed Zn^2+^ on the bare Zn electrode, which would promote the growth of zinc dendrites. The Alg@Zn anode goes through 60 s current density increase before it reaches constant. In contrast, the current density of the Alg/SiO_2_@Zn electrode reaches stabilized immediately after the polarization is applied, suggesting a homogeneous and stable 3D diffusion for Alg/SiO_2_@Zn, ensuring a uniform deposition of Zn.

The deposition morphology of the PVDF/SiO_2_@Zn symmetric cell was studied in SEM after 50 cycles. It is observed that the PVDF/SiO_2_ coating structure remains intact throughout the cycling process (Figure S24, Supporting Information). Subsequent removal of the coating layer reveals a uniformly deposited zinc foil surface, devoid of any discernible byproducts (Figure S25, Supporting Information). These findings emphasize the positive impact of SiO_2_ in the coating layer on the electrochemical performance of the Zn electrode.

Cu//Zn asymmetric cells were assembled, and the nucleation growth overpotential during the Zn deposition on modified Cu or bare Cu was investigated. The nucleation growth overpotential is calculated by the difference between the tip potential and the subsequent stable potential. The nucleation growth overpotential of Cu//Zn and Alg@Cu//Zn configuration is 50 and 45 mV, respectively (Figure 2g). Notably, the Alg/SiO_2_@Cu//Zn configuration demonstrates a further reduced nucleation growth overpotential of 38 mV. The results are consisting with the those obtained from Zn//Zn symmetric cell tests (Figure 2a). The phenomenon indicates that after introduction of SiO_2_ into the coating layer, the Zn nucleation growth barriers on Alg/SiO_2_@Cu are decreased, compared to that of Alg@Cu or bare Cu, which suggests the favorable reaction kinetics of Alg/SiO_2_ coated Zn electrode. At a current density of 2 mA cm^−2^ and an area specific capacity of 2 mAh cm^−2^, asymmetric cell of Cu//bare Zn experienced significant fluctuations after 20 cycles, which can be attributed to dendrite formation leading to short circuits (Figure 2h; Figure S26a, Supporting Information). In contrast, asymmetric cell of Alg@Cu//Zn experiences a short circuit after 75 cycles, and exhibits unstable polarization potential (Figure 2h; Figure S26b, Supporting Information). Meanwhile, asymmetric cell of Alg/SiO_2_@Cu//Zn demonstrates stable cycling beyond 105 cycles, maintaining a high Coulombic efficiency of 99.73% and a low polarization potential (Figure 2h; Figure S26c, Supporting Information).

**Figure 3:**
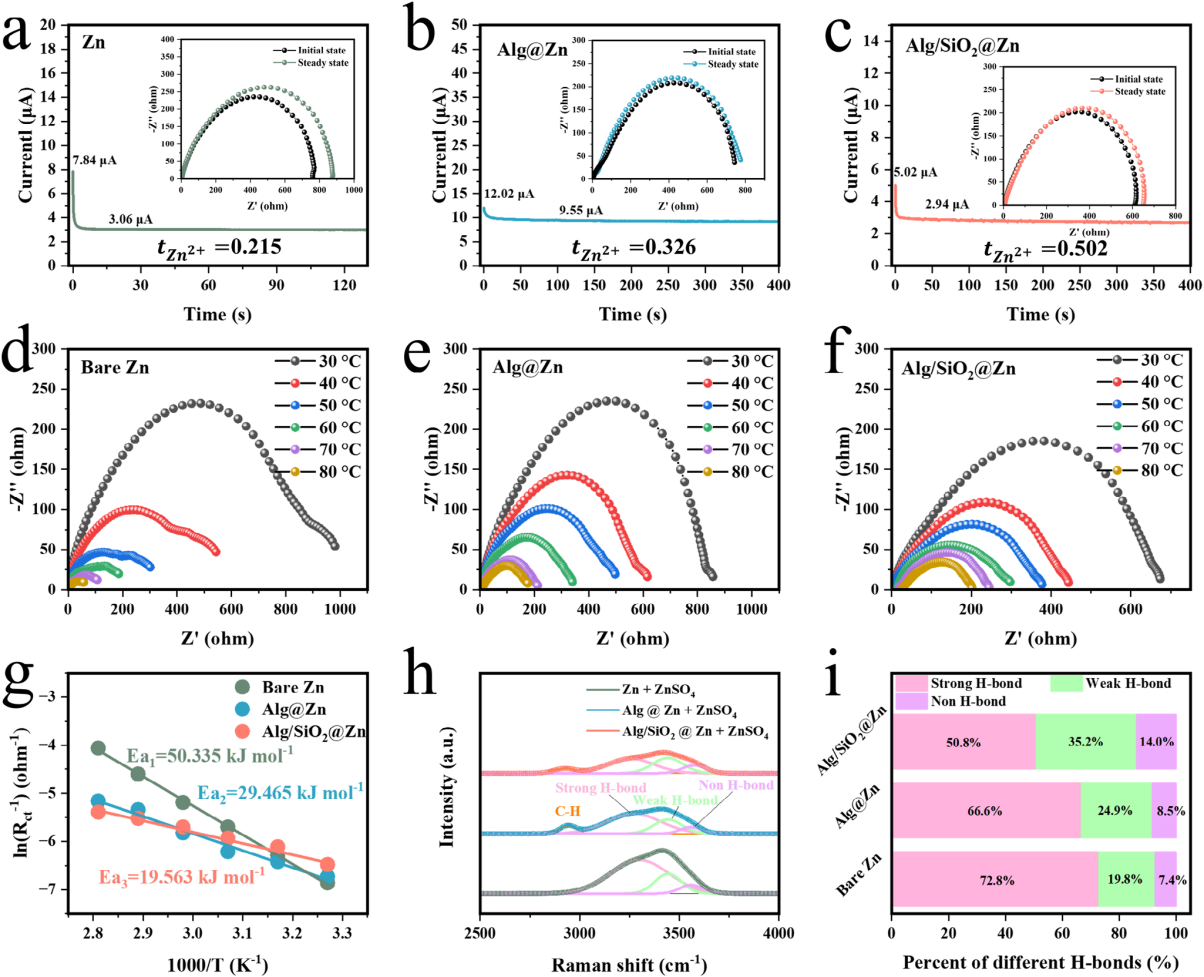
Electrochemical investigation of SiO_2_ in improving zinc ion transport kinetics. Zn^2+^ transference number of a) bare Zn, b) Alg@Zn, and c) Alg/SiO_2_@Zn. EIS at varying temperatures of d) bare Zn, e) Alg@Zn, and f) Alg/SiO_2_@Zn. g) Desolvation activation energy fitted by Arrhenius curves. h) The fitted O‐H stretching vibration represents water molecules with strong, weak, and non H‐bonds for different anodes. i) Percentage area of the three H‐bond peaks in Raman spectra.

The effect of SiO_2_ in the coating on enhancing the reaction kinetics of Zn anode is our great interest and was analyzed in detail. The Zn^2+^ transference number was first analyzed. The value of Alg/SiO_2_@Zn (0.502) (Figure 3c) is found to be higher than that of bare Zn (0.215) and Alg@Zn (0.326) (**Figure** **3**a,b). The larger Zn^2+^ transference number suggests favorable Zn^2+^ diffusion kinetics on the Zn electrode interface.

The desolvation process is often considered to be the rate‐limiting step in Zn^2+^ deposition,^[^ ^20^, ^26^ ^]^ where the large desolvation energy barrier due to the strong interaction between Zn^2+^ and the surrounding solvent H_2_O molecules slows down the reaction kinetics. During the charge transfer process, the hydrated ion [Zn(H_2_O)_6_]^2+^ present in the electrolyte would initially remove H_2_O molecules from the solvent sheath, and then migrate to the Zn surface for deposition ([Zn(H_2_O)_6_]^2+^ → Zn^2+^ + 6H_2_O).^[^ ^1b^ ^]^ To conduct a more comprehensive assessment of the impact of SiO_2_ in the coatings on the desolvation process of Zn^2+^, the activation energy (E_a_) of zinc ion transport was determined via the Arrhenius equation, employing the data obtained from Electrochemical Impedance Spectroscopy (EIS) at varying temperatures. The desolvation energy for bare Zn is E_a1_ = 50.335 kJ mol^−1^ (Figure 3d,g). And the activation energies for Alg@Zn (E_a2_) (Figure 3e,g) and Alg/SiO_2_@Zn (E_a3_) are calculated as 29.465 kJ mol^−1^ and 19.563 kJ mol^−1^, respectively (Figure 3f,g), which are much lower than that of bare Zn. The lower activation energy of Alg/SiO_2_@Zn indicates good facilitation of the Zn^2+^ desolvation process compared to bare Zn and Alg@Zn. The fast desolvation process endows it small polarization during deposition process at high current densities, favoring the fast electrochemical reaction of Zn anode. Through Raman spectroscopy analysis performed at different anode interfaces of Zn and subsequent data fitting, a significant change was observed in the hydroxyl peaks ranging from 3670 to 2940 cm^−1^ (Figure 3h), which can be divided into three sub‐peaks, attributed to strong, weak and non H‐bond, respectively. In the case of a strong hydrogen bond (H‐bond), a maximum of four H‐bonds are formed around one water molecule, forming strong H‐bond water. As to non H‐bond, there is no hydrogen bonding around the water molecules in this state, and non H‐bond water is formed. The weak H‐bond water has a number of hydrogen bonds around it between that of the strong H‐bond and non H‐bond.^[^ ^27^ ^]^ It can be seen that the strong H‐bond peak of Alg@Zn (66.6%) is lower than that of bare Zn (72.8%) (Figure 3i). The decrease of the strong H‐bond might be attributed to the interaction between the coating layer and water molecules, via which the original interaction between water molecules as well as H_2_O and Zn^2+^ in the solvation shell is disturbed, leading to lower desolvation energy.^[^ ^28^ ^]^ Interestingly, we found that the ratio of strong H‐bond of Alg/SiO_2_@Zn is further reduced to 50.8% compared to Alg@Zn, suggesting that SiO_2_ interacts with H_2_O and interrupts the hydrogen bond, which would benefit the desolvation process, thus contribute to the lowest activation energy of Alg/SiO_2_@Zn, as shown in Figure 3i.

To further investigate the effect of SiO_2_ in Alg/SiO_2_ coating on the desolvation process of zinc ions, ab initio density functional theory (DFT) calculations were carried out. Zn (002) crystal plane was chosen for calculations,^[^ ^29^ ^]^ because when the Zn is homogeneously deposited, the Zn (002) is the preferential orientation, which is observed for Alg/SiO_2_@Zn. The SiO_2_ component in Alg/SiO_2_ is amorphous, and we selected the OH‐passivated SiO_2_ (001) crystal face for the calculations, which is commonly used for calculation in literature and stable in ambient environment.^[^ ^30^ ^]^ Its surface is fully hydroxylated, which is the most stable under natural conditions. In our DFT calculations, we constructed a 3 × 2 × 2 supercell of*α‐*phase SiO_2_ and hexagonal crystalline phase zinc with 20 Å vacuum in the z‐direction. To simulate the effect of solvation environment on the deposition process of Zn atom, 32 H_2_O molecules along with a [Zn(H_2_O)_6_]^2+^ solvation structure were introduced in the vacuum region of the supercell. The DFT calculations were performed using a hybrid Gaussian and plane‐wave basis set with a local density approximation framework for the exchange correlation implemented in the cp2k code package.^[^ ^31^ ^]^ The planewave cutoff for the finest level of the multi‐grid was set as 400 Rydberg while that used for the Gaussian function was set as 60 Rydberg. A DFT‐D3 method was used to describe the van der Waals interactions between atoms. In our DFT calculations, the geometry structures were optimized until the maximum force on each atom is less than 5 × 10^−6^ Hatree per Bohr. The desolvation behavior at Zn (002) bare Zn and OH‐passivated SiO_2_ (001) coated Zn electrodes were investigated. After conducting structural analysis of the materials (Figure S27a, b, Supporting Information), we selected 3 special sites (above‐Zn, above‐deep‐Zn, between‐Zn) on the Zn (002) surface and 11 special sites (above‐O°, above‐O*, between‐O*1, between‐Si3, between‐O°1, above‐Si, between‐SC1, between‐O*3, between‐O°3, between‐SC2, and between‐Si1) on the OH‐passivated SiO_2_ (001) surface as the representative sites for further analyses (Figure S28, Supporting Information).

**Figure 4:**
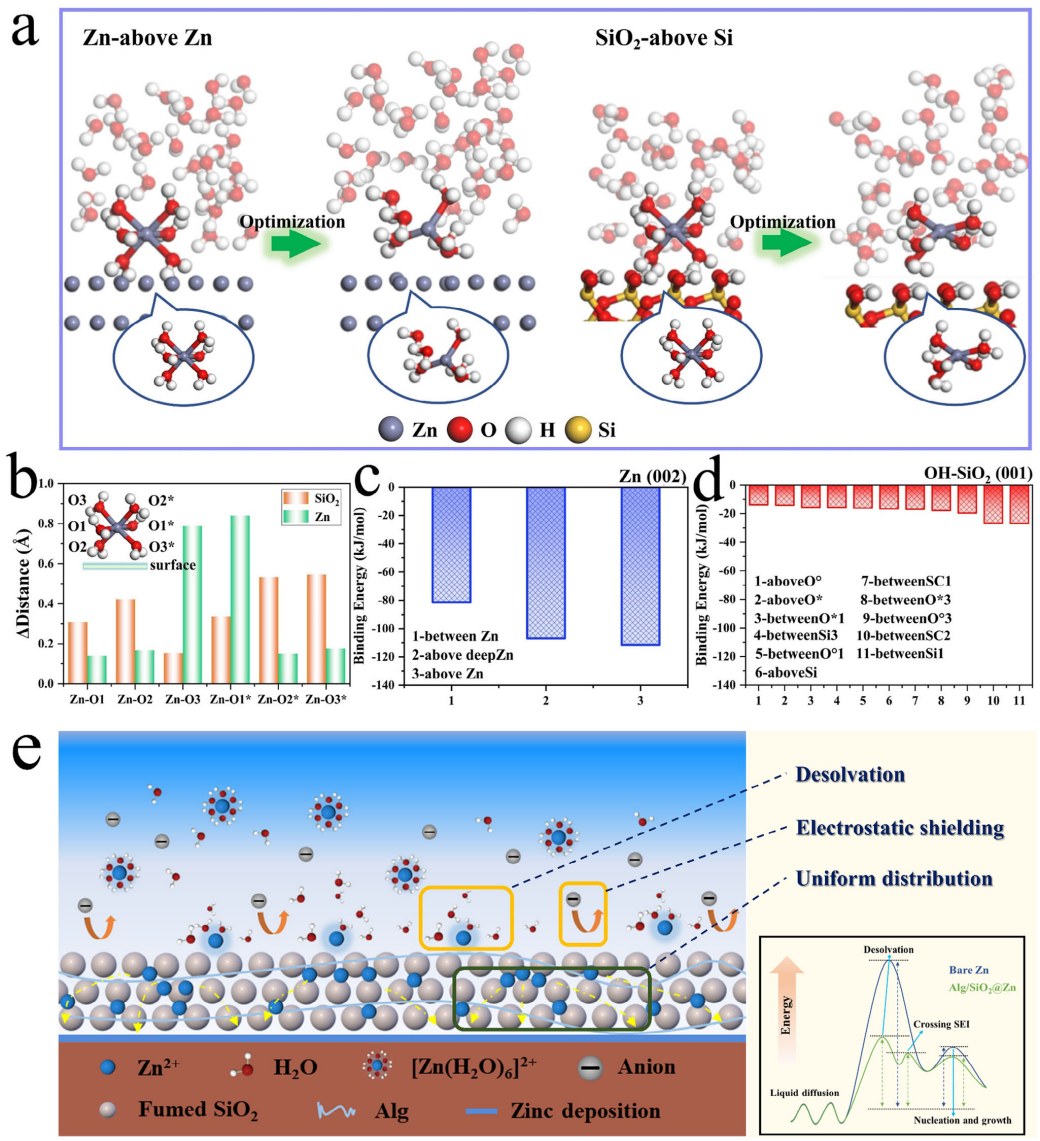
Theoretical function investigation of SiO_2_ in improving reaction kinetics and Zn^2+^ diffusion of Zn anode. a) [Zn(H_2_O)_6_]^2+^ solvation structure on Zn (002) (left) and OH‐passivated SiO_2_ (001) surface (right) surface immersed in water. The zinc, hydrogen, silicon, and oxygen atoms are presented as grey, white, yellow, and red balls, respectively. b) Average displacements between Zn and O1, O2, O3, O1*, O2*, and O3* atoms on the Zn (002) surface (green column) and OH‐passivated SiO_2_ (001) surface (orange column). A schematic diagram of the positions of Zn and O1, O2, O3, O1*, O2*, O3* is shown in the upper left picture, and the position of the surface has been marked. Adsorption energy of Zn atom at different sites of c) Zn (002) surface and d) OH‐passivated SiO_2_ (001) surface. e) Schematic illustration of the function of the Alg/SiO_2_ coating during the Zn deposition process and energy variations.

The equilibrium positions of Zn atom and H_2_O molecules on the Zn (002) and OH‐passivated SiO_2_ (001) surfaces immersed in water were first calculated. The initial (left) and optimized (right) geometries of zinc hexahydrate structures on zinc surfaces and silica surfaces are shown in **Figure** **4**a and Figure S28 (Supporting Information). To identify the diffusion of H_2_O molecules in the desolvation of [Zn(H_2_O)_6_]^2+^ structure, the oxygen atoms of the six H_2_O molecules are labelled as O1, O2, O3, O1*, O2*, and O3* (Figure S28, Supporting Information). Comparing [Zn(H_2_O)_6_]^2+^ at different positions on Zn surface (Figure S28a‐c, Supporting Information) and SiO_2_ surface (Figure S28d‐n, Supporting Information), we see the [Zn(H_2_O)_6_]^2+^ structure located at Zn (002) surface tends to keep its water sheath while the [Zn(H_2_O)_6_]^2+^ structure tends to desolvate when it locates at the OH‐passivated SiO_2_ (001) surface, especially for the case of between‐O°1, above‐Si, between‐SC1, between‐Si1, between‐Si3, between‐O*3, between‐SC1, and between‐SC2 sites, because the water molecules in [Zn(H_2_O)_6_]^2+^ structure are more dispersed. To quantitively analyze the desolvation of [Zn(H_2_O)_6_]^2+^ structure, the distances between the Zn atom and its six water sheath O atom $d_{Zn-O}^{\lambda }$ with O = O1, O2, O3, O1*, O2*, and O3* for the case of [Zn(H_2_O)_6_]^2+^ structure before (λ  =  *bef*.) and after (λ  =  *aft*.) geometry optimization were calculated. And the displacement of Zn‐O distance (Δ_*Zn* − *O*_) in the solvation structure for all sites on both Zn (002) and OH‐passivated SiO_2_ (001) surface can be determined as the difference between the distance of Zn‐O before and after optimization (Formula S1 and Table S3). The average displacement between Zn and O1, O2, O3, O1*, O2*, and O3* atoms on the Zn (002) surface (green column) and OH‐passivated SiO_2_ (001) surface (orange column) were calculated (Formula S2). As shown in Figure 4b and Table S3 (Supporting Information), the average displacements between Zn^2+^ and O3 and O1* atoms are ≈0.8 Å for the case of Zn (002) surface while the average displacements between Zn^2+^ and the other oxygen atoms are shorter than 0.2 Å. This means that H_2_O molecules with oxygen atoms at O3 and O1* sites tend to diffusion from the solvation structure while the other H_2_O molecules tend to keep the water sheath on Zn (002) surface. In contrast, for the case at the OH‐passivated SiO_2_ surface, the average displacements between Zn^2+^ and O1, O2, O1*, O2*, and O3* are larger than 0.3 Å, though that of between Zn^2+^ and O3 is smaller than 0.2 Å. This result suggests in comparison to the Zn^2+^ solvation structure at Zn (002) surface, more water molecules in the solvation structure at SiO_2_ (001) surface are inclined to desolvate, and the removal of water molecules from the solvated sheath of zinc ions on SiO_2_ surface is more preferable. To gain deep insight of the facilitated desolvation of [Zn(H_2_O)_6_]^2+^ at the OH‐passivated SiO_2_ (001) surface, the H‐bond interactions between H_2_O molecules in the water sheath and the O─H chains on the SiO_2_ (001) surface are plotted (Figure S29, Supporting Information). By measuring the bond length and bond angle of the hydrogen bonds and evaluating their similarity to standard hydrogen bonds based on the definition of hydrogen bonding (Figure S30, Supporting Information), we found that H_2_O molecules at the sites of O1, O2, and O3* in the water sheath tend to form H‐bonds with the O─H chains on the SiO_2_ surface. The results suggest that the formation of H‐bonds between water sheath and the O─H chains on SiO_2_ surface leads to the preferable desolvation of [Zn(H_2_O)_6_]^2+^ structure at the OH‐passivated SiO_2_ surface, in comparison to that at Zn surface.

The effect of SiO_2_ on regulating the flux of zinc ions is further investigated via calculating the adsorption energy of Zn atom at different sites of Zn (002) surface and OH‐passivated SiO_2_ (001) surface. The adsorption energies of Zn atom at the sites of above‐Zn, above‐deep‐Zn, and between‐Zn are −81.5, −106.9, and −111.4 kJ mol^−1^, respectively (Figure 4c). Differently, the adsorption energies of Zn atom on the OH‐passivated SiO_2_ (001) surface are in the range between −13.8 kJ mol^−1^ to −26.8 kJ mol^−1^, which are much less than those on the Zn (002) surface (Figure 4d). Moreover, we notice that the adsorption energies of Zn atom are similar for different sites on OH‐passivated SiO_2_ (001) surface. This leads to a low diffusion energy barrier of Zn atom on the OH‐passivated SiO_2_ (001) surface. Since the binding energy between Zn ions and the Zn (002) surface is larger than that between Zn ions and the OH‐passivated SiO_2_ (001), the Zn ions are more likely to be deposited on the zinc metal surface when they are deposited. At the same time, the lower transport energy barrier facilitates the uniform diffusion of Zn^2+^ between the SiO_2_‐containing coating, demonstrating the positive guiding effect of SiO_2_ on uniform deposition. On the other hand, since the energy barrier for the diffusion of Zn atom on OH‐passivated SiO_2_ (001) surface is quite low, [Zn(H_2_O)_6_]^2+^ solvation structures can easily diffuse to the sites where water sheath tends to fully collapse and desolvate into bare Zn ions. With the combined analyses with experiments and theoretical computations, the mechanism of SiO_2_ as a zinc anode coating in augmenting electrochemical performance can be displayed in Figure 4e. The Alg/SiO_2_ coating promotes the desolvation of [Zn(H_2_O)_6_]^2+^ in the solution through hydrogen bonding with water molecules. Additionally, it effectively suppresses dendrite formation by improving the migration ability of zinc ions and reducing the migration energy barrier.

**Figure 5:**
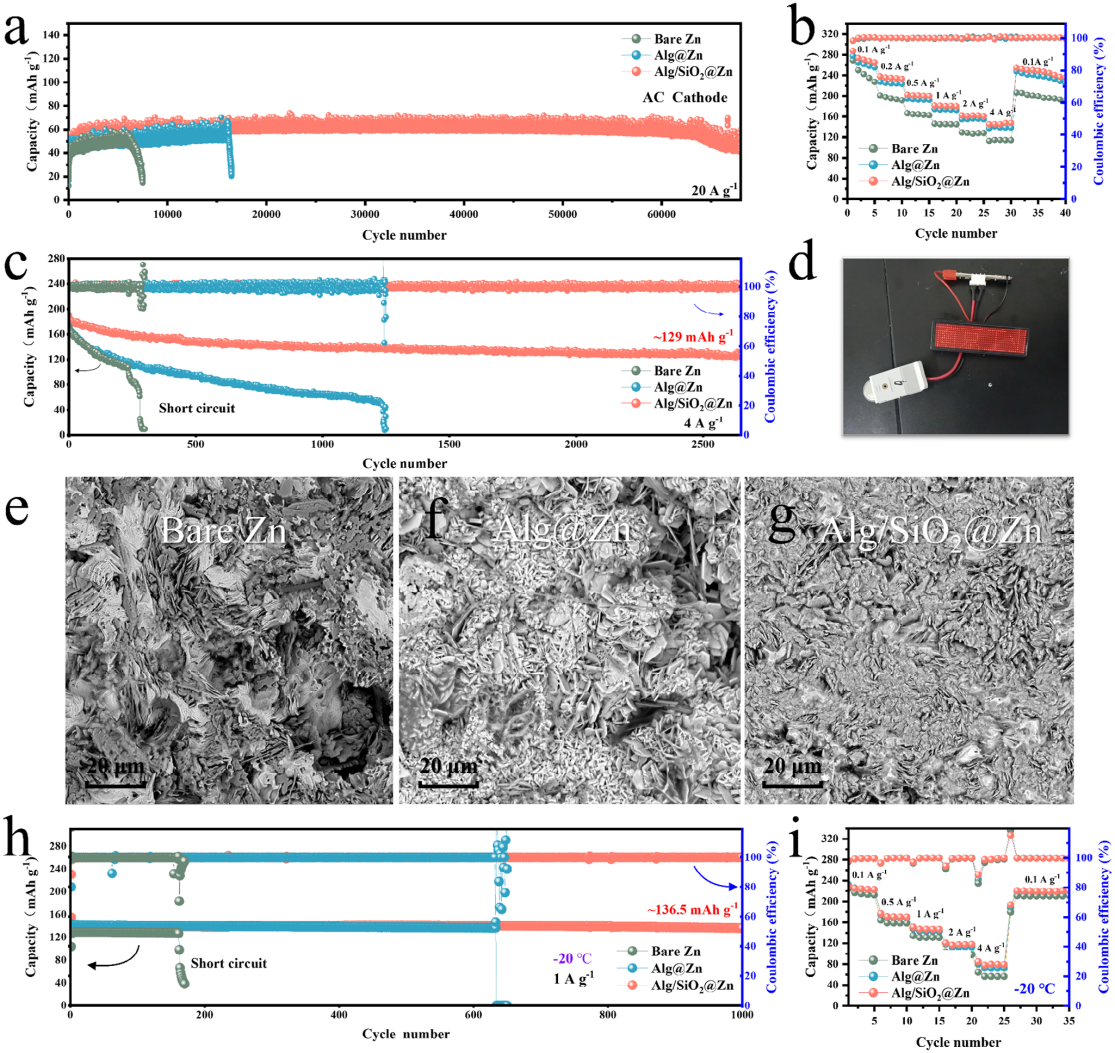
Evaluation full cells with bare Zn, Alg@Zn, and Alg/SiO_2_@Zn as anodes. a) Long‐term cycling stability of bare Zn//AC, Alg@Zn//AC, and Alg/SiO_2_@Zn//AC full cells at 20 A g^−1^. b) Rate performance of bare Zn//NVO, Alg@Zn//NVO, and Alg/SiO_2_@Zn//NVO full cells. c) Long‐term cycling stability of bare Zn//NVO, Alg@Zn//NVO, and Alg/SiO_2_@Zn//NVO full cells at 4 A g^−1^. d) The successful lighting of the light sign is achieved by connecting Alg/SiO_2_@Zn//NVO button cells in series. SEM of e) bare Zn, f) Alg@Zn, and g) Alg/SiO_2_@Zn anodes after 200 cycles in full cells with NVO as cathodes, for f) Alg@Zn and g) Alg/SiO_2_@Zn anodes, the coatings were removed. h) Long‐term cycling stability of bare Zn//NVO, Alg@Zn//NVO and Alg/SiO_2_@Zn//NVO cells were evaluated at a low temperature of −20 °C under a current density of 1 A g^−1^. i) Rate performance of bare Zn//NVO, Alg@Zn//NVO and Alg/SiO_2_@Zn//NVO full cells at a low temperature of −20 °C.

To demonstrate the feasibility of Alg/SiO_2_@Zn in practical applications, supercapacitors were assembled and evaluated with bare Zn, Alg@Zn, and Alg/SiO_2_@Zn as anodes and activated carbon (AC) as cathodes. AC was chosen as the cathode material because only the absorption/desorption process of ions occurs on the surface of AC during cycling, and influence of the cathode material can be avoided.^[^ ^16^ ^]^ Cyclic voltammetry (CV) was recorded over a voltage range of 0.2‐1.8 V (Figure S31, Supporting Information). The small redox humps observed on the CV curves may be due to faradaic redox reactions between the electrolyte and surface defects on the AC, but overall they exhibit typical capacitive behavior.^[^ ^16^ ^]^ Cycling performance was subsequently evaluated at different current densities. At a current density of 5 A g^−1^, the Alg/SiO_2_@Zn//AC exhibits around 65 000 stable cycles, whereas the bare Zn//AC and Alg@Zn//AC fail after only 3300 and 9000 cycles, respectively (Figure S32, Supporting Information). At a current density of 10 A g^−1^, the Alg/SiO_2_@Zn//AC electrode exhibits ≈34 000 stable cycles, while the bare Zn//AC and Alg@Zn//AC electrodes show short‐circuit failure at ≈7000 and 10 000 cycles, respectively (Figure S33, Supporting Information). At a current density of 20 A g^−1^, the Alg/SiO_2_@Zn//AC electrode showcases roughly 67 000 stable cycles, and the bare Zn//AC and Alg@Zn//AC electrodes experience short‐circuit failure only after approximately 7300 and 16 400 cycles, respectively (**Figure** **5**a). These results illustrate that the Alg/SiO_2_@Zn exhibits exceptional stability when compared to bare Zn and Alg@Zn. In order to further evaluate the performance of the full battery, NaV_3_O_8_·*x*H_2_O (NVO) with nanowire structure was prepared as the cathode material. The NVO cathode material was characterized by XRD and SEM (Figures S34 and S35, Supporting Information). The CV curves of NVO//Alg/SiO_2_@Zn and NVO//bare Zn full batteries at a scan rate of 1 mV s^−1^ show two sets of redox peaks are attributed to the intercalation/extraction of zinc ions into/from NVO (Figure S36, Supporting Information). For bare Zn, the two pairs of oxidation/reduction peaks located at 0.57/0.77 and 0.82/1.04 V can be ascribed to the reversible redox reactions from NaZn_0.1_V_3_O_8_·*x*H_2_O to H_2.14_NaZn_0.2_V_3_O_8_·*x*H_2_O and then H_3.9_NaZn_0.5_V_3_O_8_·*x*H_2_O, corresponding to the valence changes of vanadium from V^5+^ to V^4+^ and V^4+^ to V^3+^, respectively. In contrast, for Alg/SiO_2_@Zn//NVO cells, the two reduction peaks are at 0.59 and 0.85 V, respectively.^[^ ^31^ ^]^ The higher reduction potentials observed in the Alg/SiO_2_@Zn//NVO battery suggest that it is less polarized compared to the bare Zn//NVO battery. When tested for rate performance at current densities ranging from 0.1 to 4 A g^−1^, the Alg/SiO_2_@Zn//NVO battery demonstrates superior performance compared to the bare Zn//NVO and Alg@Zn//NVO batteries (Figure 5b). This performance improvement can be attributed to the optimization of the reaction kinetics of the zinc anode by SiO_2_ coating, and the improvement of the stability of the zinc anode. The EIS spectra of the full batteries show that Alg/SiO_2_@Zn//NVO battery exhibits the smallest impedance (Figure S37, Supporting Information), which is in line with the rate performance investigations. In a long cycle test conducted at a current density of 4 A g^−1^, the Alg/SiO_2_@Zn//NVO full cell demonstrated stability for 2650 cycles with 67.9% capacity retention (Figure 5c). In comparison, the bare Zn//NVO and Alg@Zn//NVO full cells experience short‐circuiting and fail after 300 and 1250 cycles, respectively. The zinc anode after circulation was further observed by SEM. The bare Zn surface displays noticeable dendrite formation and uneven deposition surfaces, accompanied by the presence of a significant quantity of distributed by‐products (Figure 5e). For the Alg@Zn//NVO battery, dendrites can also be found after cycling at the surface of the Zn when the coating is removed (Figure 5f), which is consistent with our previous reports.^[^ ^16^ ^]^ Conversely, upon the removal of the Alg/SiO_2_ coating, the Zn exhibits a uniformly deposited surface with no obvious dendrites or by‐products (Figure 5g). The above analyses show that the Alg/SiO_2_ coating inhibits the occurrence of side reactions on the zinc anode surface while facilitating the desolvation process of hydrated zinc ions, providing faster zinc ion transport kinetics, and guiding the uniform deposition of zinc ions. The light sign can be successfully powered by connecting Alg/SiO_2_@Zn//NVO cells in series, demonstrating of the potential application of Alg/SiO_2_@Zn as a promising anode for AZIBs (Figure 5d). To further highlight the positive role of SiO_2_ in the coating layer, PVDF/SiO_2_@Zn//NVO full battery was prepared and subjected to testing as a control. The battery demonstrates favorable cycling stability at a current density of 4 A g^−1^. Impressively, even after 1240 cycles, the discharge specific capacity remains at 100 mAh g^−1^, indicating a significantly improved performance compared to the bare Zn//NVO full cell (Figure S38, Supporting Information). In addition, when examining the Zn foil after removal of the PVDF/SiO_2_ coating through SEM after 200 cycles, only a small amount of dendrite formation was observed, suggesting a substantial enhancement compared to the bare Zn configuration (Figure S39, Supporting Information).

The performance of full batteries with different Zn anodes and NVO cathode was further studied at a low temperature of −20 °C, with 3 m Zn(CF_3_SO_3_)_2_ as electrolyte. It is widely recognized that electrochemical reaction kinetics are impeded at lower temperatures, resulting in an amplified polarization effect. This elevated polarization, in turn, promotes the formation of dendrites, which can severely compromise the lifespan of the battery.^[^ ^32^ ^]^ The cycling performance of bare Zn//NVO, Alg@Zn//NVO, and Alg/SiO_2_@Zn//NVO full cells was tested at a current density of 1 A g^−1^. The Alg@Zn//NVO battery short‐circuits after 630 cycles, and the bare Zn//NVO battery could only be cycled for 160 times. In contrast, the Alg/SiO_2_@Zn//NVO battery exhibits excellent long cycling stability, with a discharge specific capacity of 136.5 mAh g^−1^ after 1000 cycles (Figure 5h). Compared with the currently reported AZIBs with vanadium‐based cathode materials applied to low temperatures, the cycle life of the full battery assembled by our SiO_2_‐coated zinc anode still shows significant advantage (Table S6, Supporting Information). In addition, Alg/SiO_2_@Zn//NVO battery exhibits best rate performance, when a current density of 4 A g^−1^ is applied, a capacity of 80 mAh g^−1^ can still be achieved. (Figure 5i)

## 3 Conclusion

In conclusion, Alg/SiO_2_ coatings with high Young's modulus, high electrolyte wettability, and abundant negative charge were prepared in situ on Zn metal surfaces by a cost‐effective scraper method. Combined spectral analysis and theoretical calculations show that the abundant functional groups on the surface of SiO_2_ facilitate the desolvation process of hydrated zinc ions through hydrogen bonding with water molecules. And the kinetics of zinc deposition process is greatly improved. In addition, the low binding energy between Zn^2+^ and SiO_2_ can provide a fast transport path and uniform flux distribution for Zn^2+^, and guide the preferential Zn deposition along (002) plane. Furthermore, the abundant negative charge from SiO_2_ has a strong electrostatic repulsive effect on the SO_4_^2−^ anion, effectively inhibiting self‐corrosion at the interface and providing high Zn^2+^ transference number. As a result, the Alg/SiO_2_@Zn symmetric cell can be stably cycled for ≈3000 h at a current density of 1 mA cm^−2^ and a surface capacity of 1 mAh cm^−2^. At a current of 10 mA cm^−2^, the symmetric cell could be stably cycled for 340 h. Capacitor assembled with Alg/SiO_2_@Zn anode and AC cathode achieves ultra‐stable cycles of over 67 000 cycles at a current density of 20 A g^−1^. For a full cell with Alg/SiO_2_@Zn anode and NVO cathode, a long cycle life of 2650 cycles at a current density of 4 A g^−1^ and a capacity retention of 67.9% is achieved. The cell has a discharge capacity of 136.5 mAh g^−1^ after 1000 cycles at the extreme temperature of −20 °C.

## Conflict of Interest

The authors declare no conflict of interest.

## Supporting information



Supporting Information

## Data Availability

The data that support the findings of this study are available in the supplementary material of this article.
