# Supplementary material for: Favorable Desolvation and Uniform Zn Deposition of Silica Modified Zn Anode for High Performance Aqueous Zn‐Ion Batteries
Source: Adv Sci (Weinh). 2025 Apr 2;12(22):2417121. doi: 10.1002/advs.202417121 (PMC12165026; doi:10.1002/advs.202417121)
Supplement: Supplementary file 1 — Supporting Information [file ADVS-12-2417121-s001.docx]

**Supporting Information**

**Favorable Desolvation and Uniform Zn Deposition of Silica Modified Zn Anode for High Performance Aqueous Zn-ion Batteries**

Wangsheng Yuan^+[a]^, Kai Jin^+[b]^, Shanbao Zou^[a]^, Yishan Jin^[a]^, Yishuang He^[a]^, Xinhai Yuan^[a]^, Peng Han*^[b]^, Lijun Fu*^[a]^, Yuping Wu^[a]^

[a] State Key Laboratory of Materials-Oriented Chemical Engineering, School of Chemistry and Molecular Engineering and College of Energy Science and Engineering, Nanjing Tech University, Nanjing, Jiangsu, 211816, China.

Email: l.fu@njtech.edu.cn; [lijunfufu@sina.com](mailto:lijunfufu@sina.com) (L. Fu)

[b] Department of Physics, Capital Normal University, Beijing, 100048, China.

Email: [peng.han@cnu.edu.cn](mailto:peng.han@cnu.edu.cn) (P. Han)

EXPERIMENTAL SECTION

**Preparation of** **Alg/SiO_2_@Zn and Alg@Zn.** Sodium alginate powder was mixed with hydrophilic fumed silica (SiO_2_) powder at a mass ratio of 1:1, then deionised water was added at a mass ratio of 1:50 with sodium alginate and sealed in a glass bottle and stirred for 24 h at room temperature to obtain a homogeneous viscous mixture. A commercial zinc foil with a purity of 99.99% and a thickness of 100 μm was sanded and cleaned on both sides, then cut to 1 cm × 1 cm.

The mixture was aspirated using a pipette gun and added dropwise to the already treated zinc foils and the mixture was evenly coated using a spatula. The zinc foils were then immersed in 2 M ZnSO_4_ aqueous solution and waited for 30 s. Zinc foils coated with zinc alginate/ hydrophilic fumed silica (Alg/SiO_2_@Zn) were prepared in situ. Similarly, zinc foils coated with zinc alginate (Alg@Zn) were prepared without the addition of SiO_2_.

**Preparation of PVDF/SiO_2_@Zn and PVDF@Zn.** To prepare PVFD/SiO_2_@Zn anode, the PVFD/SiO_2_ slurry was prepared by mixing SiO_2_ powder and Polyvinylidenefluoride (PVFD) in a weight ratio of 1:1 by 1-methyl-2-pyrrolidone (NMP). Then, the slurry was coated on Zn foil and dried at 60 ℃ for 12 h. Similarly, PVDF coated zinc foils (PVDF@Zn) were prepared without the addition of SiO_2_.

**Preparation of NVO cathode material.** 3 g of commercial vanadium pentoxide (V_2_O_5_) and 11.7 g of sodium chloride (NaCl) were mixed and added to 100 ml of deionised water, heated at 30 °C and stirred for 72 h. The solution was then washed by filtration and the precipitate was dried in an oven at 60 °C for 12 h to give NaV_3_O_8_·*x*H_2_O (NVO) powder.

**Materials Characterizations.** The crystallographic structure was analyzed by X-ray diffractometer (XRD, D/MAX-IIA, Rigaku) with Cu-Kαradiation (λ = 0.15406 nm) at a scanning angle (2θ) range of 5° to 90°. The morphology and microstructure observation of the samples was measured by scanning electron microscopy (SEM), energy-dispersive spectrometer (EDS, XL-70, Philips) and optical microscope (YM520R). Raman spectroscopy was conducted on Horiba LabRAM HR Evolution microscope with a 532 nm excitation laser.

**Electrochemical Measurements.** Symmetrical batteries were tested using a glass fiber separator with Zn and improved anodes (Alg/SiO_2_@Zn, Alg@Zn, PVDF/SiO_2_@Zn and PVDF@Zn) in a CR2025 coin battery and assembled in the air. 2 M ZnSO_4_ is used as electrolyte at room temperature and 3 m Zn(CF_3_SO_3_)_2_ at -20 ℃ as electrolyte. The electrolyte content is 60 μL.

After that, full cells were fabricated with Bare Zn, Alg/SiO_2_@Zn and AC/NVO. AC was mixed with Carbon Black (CB), and Polytetrafluoroethylene (PTFE) at a weight ratio of 8: 1: 1, and then the slurry dried under infrared baking lamp for 1 h. The mixture is then rolled into a uniform sheet and dried in an oven at 60 ℃ for 12 h. The mass of cathode is about 4 mg cm^-2^. The NVO, Carbon Black, and PTFE binder were grinded at a mass ration of 7:2:1. The viscous mixture dried under infrared baking lamp for 1 h. The mixture is then rolled into a uniform sheet and dried in an oven at 60 ℃ for 12h. The mass of cathode is about 4 ~ 8 mg cm^-2^. All cells use stainless steel mesh as the collector and glass fiber (GF/A) as the separator.

The electrochemical performance was implemented by a battery test system (LAND). Nucleation overpotential and coulombic efficiency (CE) measurements that reflect the reversibility of the anode were performed on asymmetrical Zn||Cu cells in 2 M ZnSO_4_. The Cyclic voltammetry (CV) was recorded on an electrochemical workstation (CHI660E). The scanning speed is 1 mV s^-1^. Chronoamperogram (CA) measurements of Bare Zn and Alg/SiO_2_@Zn were carried out at a constant potential of -150 mV. Electrochemical Impedance Spectroscopy (EIS) was measured by an impedance/gain-phase analyzer (Solartron analytical 1260A) and an electrochemical test analyzer (Solartron analytical 1287A). EIS spectra were recorded over the frequency ranging from 100 kHz to 0.01 Hz with an amplitude of 10 mV.

Zn^2+^ transference number ($t_{{Z_{n}}^{2+}}$) was evaluated by the following equation:

$t_{{Z_{n}}^{2+}}=\frac{I_{s}\left( \Delta V-I_{0}R_{0} \right)}{I_{0}\left( \Delta V-I_{s}R_{s} \right)}$ (1)

where $\Delta V$ is the constant polarization voltage applied (10 mV), $I_{0}$ and $R_{0}$ are the initial current and resistance, $I_{s}$ and $R_{s}$ are the steady-state current and resistance, respectively.

Desolvation activation energies ($E_{a}$) were evaluated by measuring EIS of Zn//Zn symmetrical cell at different temperatures, the $E_{a}$ can be described by the law of Arrhenius equation:

$\frac{1}{R_{ct}}=Aⅇxp\left( -\frac{E_{a}}{RT} \right)$ (2)

where $R_{ct}$ is the charge transfer resistance, $A$ is the frequency factor, $R$ is the gas constant and $T$ is the absolute temperature.

**Ab initio density functional theory (DFT) Calculations**

Ab initio density functional theory (DFT) is the state of the art working horse for calculating the physical and chemical properties of many particle systems from molecules to condensed matters. By separating the fast moving electrons and the slowly moved nuclei based on the idea of Born-Oppenheimer approximation, any property of a system composed by many interacting particles is viewed as a functional of the ground state electron density in the framework of ab initio DFT. Using this approach, the total energy of a geometry optimized many particles system can be accurately calculated without using any empirical parameters except basic physical quantities such as masses and charges.

**Computational Details**

We perform ab initio density functional theory (DFT) calculations to study the adsorption energy of Zn atom and the desolvation of [Zn(H_2_O)_6_]^2+^ structure at Zn (002) surface and OH-passivated SiO_2_ (001) surface. In our calculation, we construct a 3×2 supercell of Zn atoms with hexagonal structure in the xy direction and four atomic layers in the z direction. To mimic Zn (002) surface, a vacuum layer of 20 Å is introduced in the z direction. Similar to the Zn (002) surface, the OH-passivated SiO_2_ (001) surface is constructed by a 3×2×2 supercell of α-phase SiO_2_ with 20 Å vacuum in the z direction. To passivate the surface dangling bonds in the z direction, we remove Si atoms with only two nearest neighboring O atoms and terminate the dangling bond of O atom by a hydrogen atom.

To calculate the adsorption energy of Zn atom, a bare Zn atom is introduced on different sites of Zn (002) and OH-passivated SiO_2_ (001) surfaces. The equilibrium positions of atoms are obtained until the force on each atom reduced to less than 1×10^-5^eV/Å. The adsorption energy E_abs_ is then calculated via equation:

E_abs_=E(surface+Zn) - E(surface) - E(Zn) (3)

where, E(surface+Zn), E(surface), and E(Zn) denote the total energy of the Zn (or SiO_2_) surface with absorbent Zn atom, the energy of the Zn (or SiO_2_) surface and the energy of a Zn atom, respectively.

To mimic the desolvation of [Zn(H_2_O)_6_]^2+^ structure in water solution, we introduce H_2_O molecules in the vacuum layer of the Zn (002) and OH-passivated SiO_2_ (001) supercells with density of 0.95 g/ml. The snapshots of Zn (002) and OH-passivated SiO_2_ (001) supercells with [Zn(H_2_O)_6_]^2+^ structure in water solution are given (Figure S28). The structural changes of zinc hexahydrate at other sites are recorded, where the water molecules have been hidden.

To obtain the equilibrium positions of Zn atom and H_2_O molecules, i.e. the desolvation of [Zn(H_2_O)_6_]^2+^ structure at the Zn (002) and OH-passivated SiO_2_ (001) surfaces in water solution, we optimize the positions of Zn atom and H_2_O molecules (both in the [Zn(H_2_O)_6_]^2+^ structure and free water molecules in solution) until the force on each atom reduced to less than 1×10^-5^ eV/Å with fixing the positions of atoms in the Zn (002) and OH-passivated SiO_2_ (001) surfaces.

All the DFT calculations are performed using the CP2K code package^[1]^ within the framework of local density approximation functional and a hybrid Gaussian and plane-wave scheme. The molecular orbitals of the valence electrons used in the calculation are expanded into the DZVP-GTH-PADE basis sets^[2]^ and the place waves are expanded with a cutoff of 400 Rydberg. In our ab initio calculation, we use the Goedecker-Teter-Hutter (GTH) pseudopotentials to describe the atomic core electrons and the DFT-D3 method to correctly describe van der Waals interactions^[3]^.

**Structural analysis and selection of special sites**

For Zn surfaces, we chose the (002) crystal orientation because the (002) orientation of Zn is the primary crystal orientation of the electrode after battery cycling. We designate the zinc atom positioned in the surface layer as the "Zn" atom, and the zinc atom found in the second layer as the "deep Zn" atom. The zinc and deep zinc atoms presented as grey and black balls in order to see more clearly (Figure S27a). To systemically study the adsorption energy of Zn atom at Zn (002), we select three different sites, i.e., above-Zn where the additional Zn atom locates above a Zn atom at the (002) surface, above-deep-Zn where the additional Zn atom locates above a Zn atom in a deep layer of the (002) surface, and between-Zn where the deposited Zn atom locates between two Zn atoms at the (002) surface, in our calculation. The top view of these sites is given with the zinc and deep zinc atoms presented as gray and black balls in order to see them more clearly (Figure S27b). The dot represents above the atom and the line represents between the two atoms. The initial position of all special sites is 3Å above the interface. We performed structural optimization before performing the binding energy calculation. For OH-passivated SiO_2_ (001) surfaces, we designate the oxygen atom at the higher position as the O* (Star) atom and the oxygen atom at the lower position as the O° (Cycle) atom (Figure S27a). For the case of OH-passivated SiO_2_ (001) surface, we selected 11 different sites to cover all the special positions on the OH-passivated SiO_2_ (001) surfaces，i.e. above-O°, above-O*, between-O*1, between-Si3, between-O°1, above-Si, between-SC1, between-O*3, between-O°3, between-SC2, and between-Si1. 1 represents the nearest neighbor atom, 3 represents the second nearest neighbor atom. The top view of these sites is given (Figure S27b), with the hydrogen, oxygen, and silicon atoms presented as white, red, and yellow balls, respectively. The definition of the O° and O* atom are also given (Figure S27b).

**Formula S1.** Formula for calculating distance variation.

$\Delta_{Zn-O}=|d_{Zn-O}^{bef.}-d_{Zn-O}^{aft.}|$ (4)

where $d_{Zn-O}^{bef.}$ is the distance between oxygen atoms and zinc before; $d_{Zn-O}^{aft.}$ is the distance between oxygen atoms and zinc after structural optimization.

**Formula S2.** Formula for calculating the average change.

$\bar{\Delta_{\mathrm{Zn}-O}}=\frac{\sum_{i=1}^{n} \Delta_{\mathrm{Zn}-O}^{i}}{n}$ (5)

where $n$ is the number of sites on each surface; $\Delta_{\mathrm{Zn}-O}^{i}$ is displacement of the $i^{th}$site.

**Table S1.** Performance comparison of Alg/SiO_2_@Zn and those Zn electrode with coating modification in literature.

| **Strategies** | **Current density**  **(mA cm^-2^)** | | **Areal capacity**  **(mAh cm^-2^)** | | | **Polarization**  **（mV）** | | **Cycle time (h)** | **Reference** | |
| --- | --- | --- | --- | --- | --- | --- | --- | --- | --- | --- |
| ZF@F-TiO_2_ | | 1 | | 1 | 28 | | 480 | | | ^[4]^ |
| (100) facet TiO_2_ | | 1 | | 1 | 42 | | 130 | | | ^[5]^ |
| In@Zn | | 1 | | 1 | 32 | | 300 | | | ^[6]^ |
| NaTi_2_(PO_4_)_3_ | | 1 | | 1 | 40 | | 130 | | | ^[7]^ |
| ZnO@Zn | | 1 | | 1 | 54 | | 400 | | | ^[8]^ |
| PVDF/Al_2_O_3_-25@Zn | | 1 | | 0.5 | 25 | | 300 | | | ^[9]^ |
| Ag-Zn | | 1 | | 1 | 28 | | 350 | | | ^[10]^ |
| Se_2_O_3_@Zn | | 1 | | 1 | 34 | | 200 | | | ^[11]^ |
| 3D ZnF_2_@Zn | | 1 | | 1 | 71.5 | | 800 | | | ^[12]^ |
| ZnF_2_@Zn | | 1 | | 1 | 60 | | 2500 | | | ^[13]^ |
| Zn_3_(PO_4_)@Zn | | 1 | | 1 | 80 | | 1200 | | | ^[14]^ |
| 502 glue@Zn | | 0.5 | | 0.25 | 100 | | 800 | | | ^[15]^ |
| IZIF-8@Zn | | 1 | | 1 | 100 | | 1400 | | | ^[16]^ |
| Alucone | | 1 | | 1 | 498 | | 46.1 | | | ^[17]^ |
| SiO_2_@Zn | | 1 | | 0.5 | 50 | | 1000 | | | ^[18]^ |
| **This Work** | | **1** | | **1** | **24** | | **2950** | | | / |
| Zn@ZnO/C-Cu | | 5 | | 1 | 120 | | 530 | | | ^[19]^ |
| HZE-Zn | | 2 | | 5 | 50 | | 300 | | | ^[20]^ |
| **This Work** | | **5** | | **5** | **36.5** | | **840** | | | / |
| Zn@ZIF8 | | 10 | | 5 | 110 | | 220 | | | ^[21]^ |
| Zn@ZnP | | 10 | | 10 | 60 | | 95 | | | ^[22]^ |
| Polyamide@Zn | | 10 | | 10 | 100 | | 75 | | | ^[23]^ |
| ZnMoO_4_-PVA@SR | | 10 | | 10 | 75 | | 250 | | | ^[24]^ |
| DCP-Zn-30@Zn | | 5 | | 10 | 70 | | 200 | | | ^[25]^ |
| **This Work** | | **10** | | **10** | **60** | | **380** | | | / |

**Table S2.** Elemental composition and Zn/Si ratio of Alg/SiO_2_ coatings

before and after 50 cycles (100 h).

| **Atomic**  **percentage** | **Zn** | **Si** | **C** | **O** | **Zn/Si** |
| --- | --- | --- | --- | --- | --- |
| Before deposition | 7.91% | 22.17% | 19.19% | 50.74% | 2.803 |
| After deposition | 4.18% | 11.94% | 25.85% | 58.03% | 2.856 |

**Table S3.** Average displacements between Zn and O1, O2, O3, O1*, O2*, and O3* atoms on the Zn (002) surface and OH-passivated SiO_2_ (001) surface.

|  | **ΔDistance (Å)**  **(OH-passivated SiO_2_ (001) surface)** | **ΔDistance (Å)**  **(Zn (002) surface)** |
| --- | --- | --- |
| Zn-O1 | 0.30808 | 0.14 |
| Zn-O2 | 0.42271 | 0.167 |
| Zn-O3 | 0.15337 | 0.788 |
| Zn-O1* | 0.33535 | 0.84 |
| Zn-O2* | 0.53302 | 0.15067 |
| Zn-O3* | 0.54543 | 0.17567 |

**Table S4.** The binding energies of Zn at different sites on the Zn surface.

| **Position** | **Binding Energy (kJ mol^-1^)** |
| --- | --- |
| Between Zn | -81.4946 |
| Above deep Zn | -106.91674 |
| Above Zn | -111.44365 |

**Table S5.** The binding energies of Zn at different sites on the OH-passivated SiO_2_ (001) surface.

| **Position** | **Binding Energy (kJ mol^-1^)** |
| --- | --- |
| Above O° | -13.75914 |
| Above O* | -14.03717 |
| Between O*1 | -15.58744 |
| Between Si3 | -15.64663 |
| Between O°1 | -16.1306 |
| Above Si | -16.6951 |
| Between SC1 | -16.97855 |
| Between O*3 | -17.82692 |
| Between O°3 | -19.68082 |
| Between SC2 | -26.6871 |
| Between Si1 | -26.83494 |

**Table S6.** Low-temperature performance comparison of AZIBs with vanadium-based cathode materials under various modification strategies.

| **Strategies** | **Key improvement point** | **Basic Electrolyte** | **Cycling Performance** | **Working temperature** | | **Reference** | |  |
| --- | --- | --- | --- | --- | --- | --- | --- | --- |
| Organic additive | N, N-dimethyl acetamide | 2 M ZnSO_4_ | 1 A g^−1^ for 500 cycles | | -18°C | | ^[26]^ | |
| Organic additive | 10 vol% ethylene glycol | 3 M Zn(CF_3_SO_3_)_2_ +0.1 M ZnI_2_ | 0.2 A g^−1^ for 200 cycles | | -20°C | | ^[27]^ | |
| Organic additive | 50 vol% trimethyl phosphate | 2 M Zn (CF_3_SO_3_)_2_ | 1 A g^−1^ for 1200 cycles | | 0°C | | ^[28]^ | |
| Gel electrolyte | Glycerol/Acetonitrile | 3 M ZnSO_4_ | 0.5 A g^−1^ for 500 cycles | | -20°C | | ^[29]^ | |
| Anode interface modification | Layered zinc silicate nanosheet (Zn@LZS) | 3 M Zn(CF_3_SO_3_)_2_ | 1 A g^−1^ for 200 cycles | | 0°C | | ^[30]^ | |
| **Anode interface modification** | **This Work** | **3 m Zn(CF_3_SO_3_)_2_** | **1 A g^−1^ for 1000 cycles** | | **-20°C** | | **/** | |

**
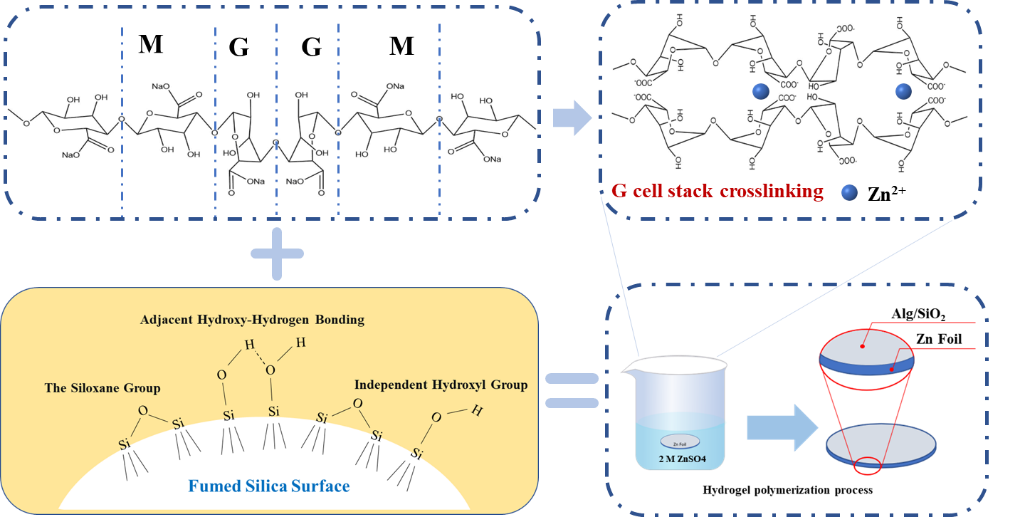
**

**Figure S1.** Diagram of the Alg/SiO_2_@Zn preparation process.

**
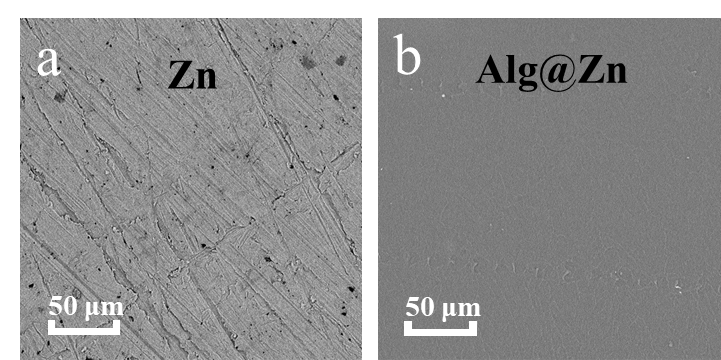
**

**Figure S2.** SEM of (a) bare Zn and (b) Alg@Zn before cycling.


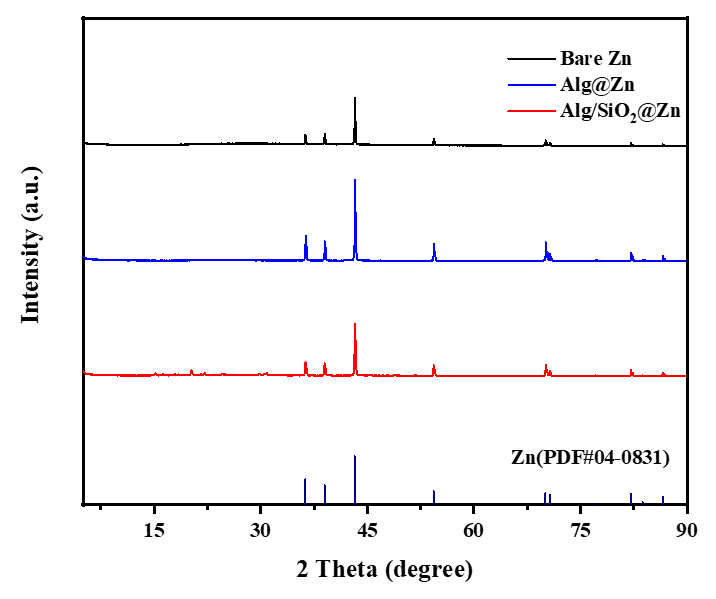


**Figure S3.** XRD of bare Zn, Alg@Zn and Alg/SiO_2_@Zn before cycling.

**
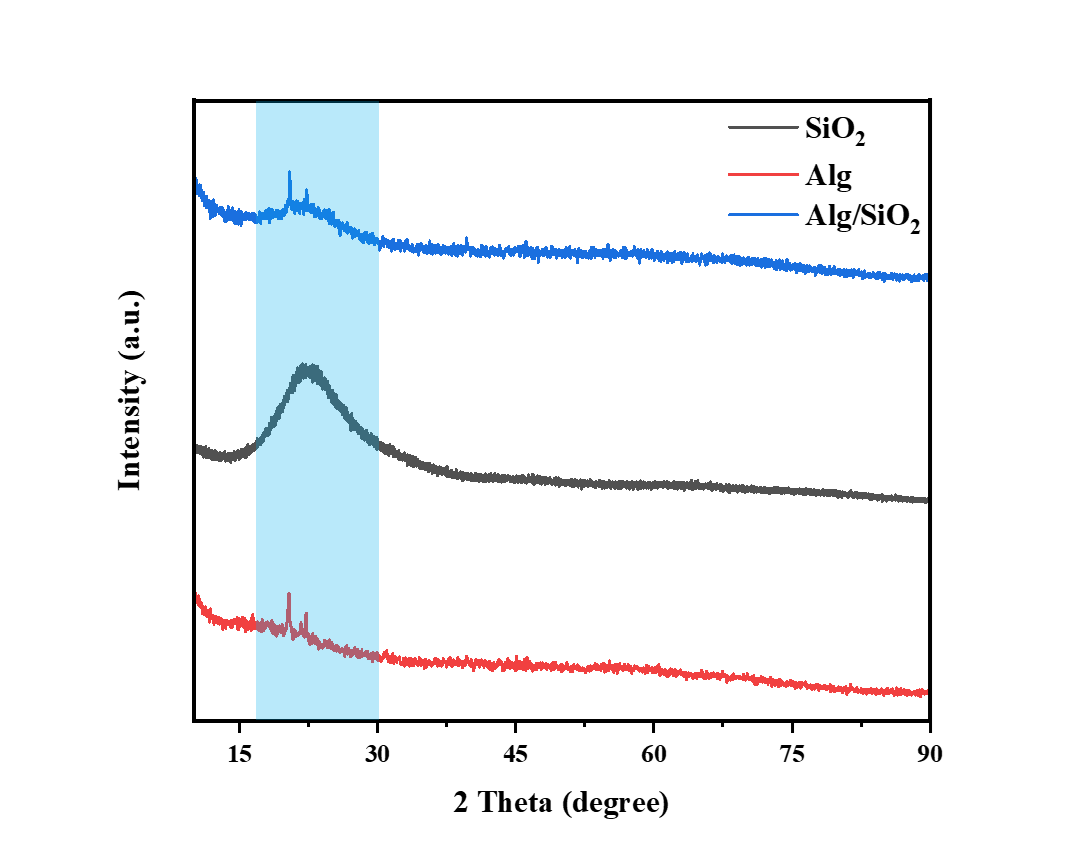
**

**Figure S4.** XRD of SiO_2_ powder, Alg and Alg/SiO_2_ coatings.

**
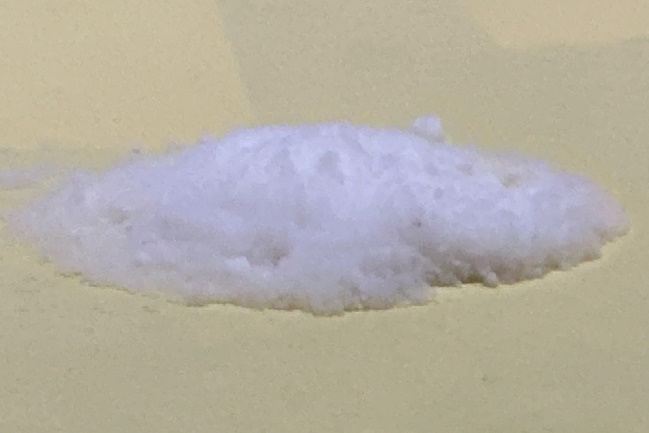
**

**Figure S5.** Optical photograph of hydrophilic nano-fumed SiO_2_ powder.

**
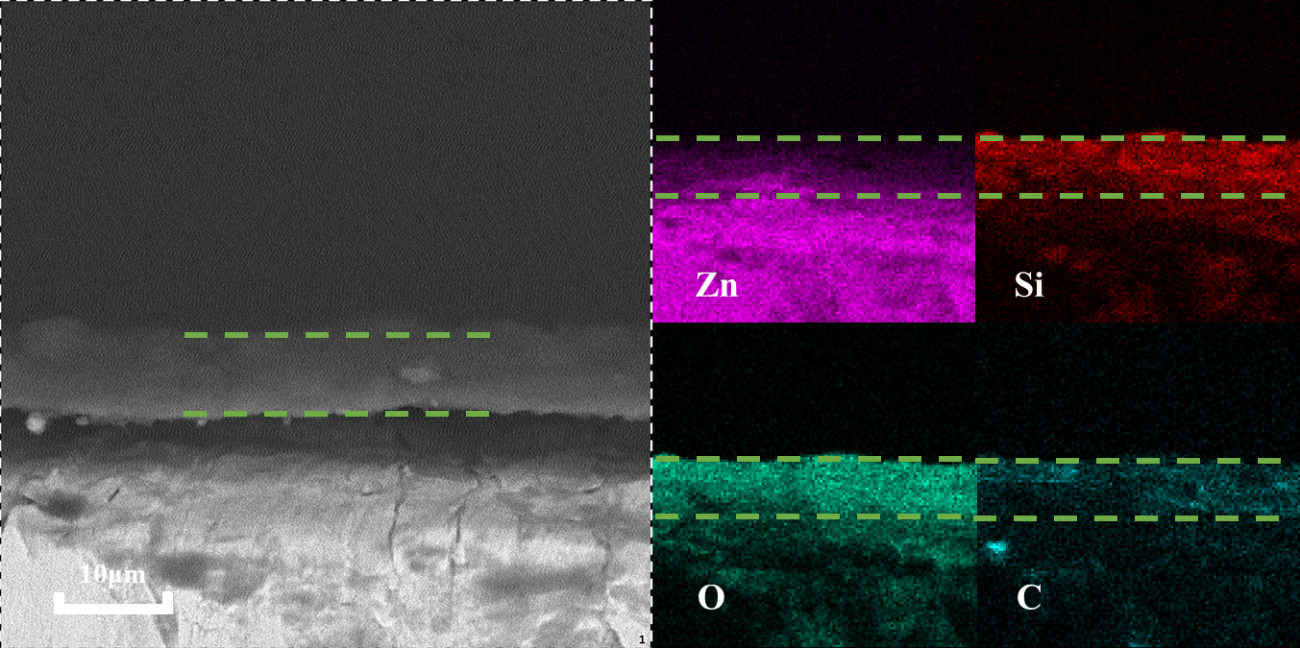
**

**Figure S6.** EDS of the Alg/SiO_2_@Zn section before cycling.

**
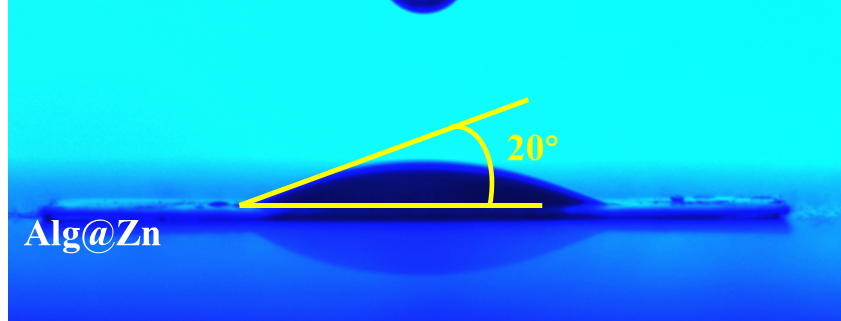
**

**Figure S7.** Contact angle measurements for Alg@Zn.

**Self-corrosion resistance test**

It is commonly believed that in a mildly acidic electrolyte, the side reaction of hydrogen evolution on the surface of Zn metal leads to an increase in the local OH^−^ concentration, and the reaction between OH^−^, H_2_O, Zn^2+^ and SO_4_^2−^ leads to the formation and precipitation of Zn_4_SO_4_(OH)_6_·H_2_O by-products, as demonstrated in previous reports. To investigate the inhabitation of self-corrosion for the modified Zn anodes, bare Zn and zinc foils covered with different coatings were immersed in the 2 M ZnSO_4_ electrolyte for 7 days. SEM analyses reveal that for the bare Zn, substantial flakes of by-products are present after a 7 days soaking period (Figure S8a, d), and the XRD investigation confirms the existence of Zn_4_SO_4_(OH)_6_·H_2_O (Figure S9). In contrast, no significant presence of Zn_4_SO_4_(OH)_6_·H_2_O by-products is observed for Alg@Zn (Figure S8b, e) and Alg/SiO_2_@Zn (Figure S8c, f). Additionally, the intensity of the XRD diffraction peak associated with the by-products is decreased as well for modified Zn (Figure S9). Notably, the diffraction peak intensity of the by-products in Alg/SiO_2_@Zn is further reduced compared to Alg@Zn. Upon examining the SEM of the Zn metal surface after removing the Alg/SiO_2_ coating (Figure S10), it closely resembles bare Zn prior to immersion, with no appearance of any by-products. This indicates that the addition of SiO_2_, the Alg/SiO_2_ coating effectively inhibits the self-corrosion of the Zn anode, and providing reliable protection for the Zn anode.

**
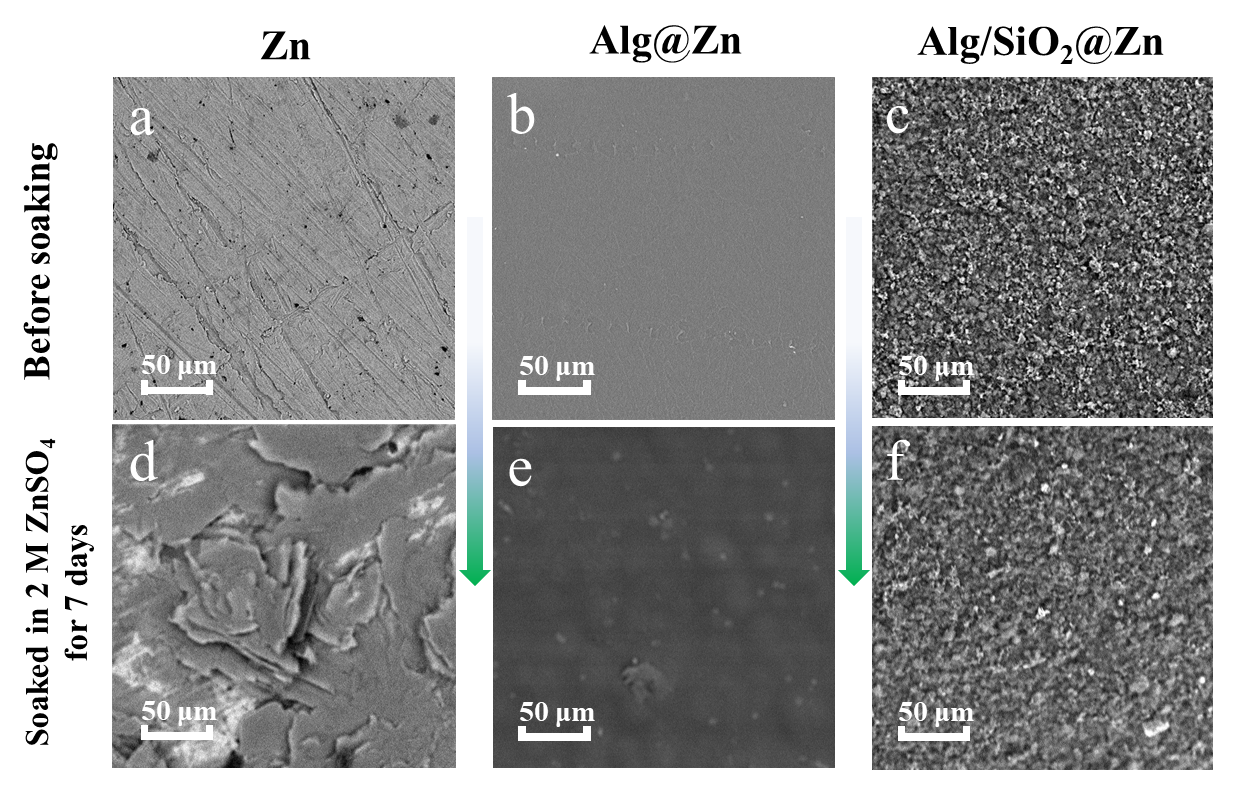
**

**Figure S8.** SEM of (a, d) bare Zn, (b, e) Alg@Zn and (c, f) Alg/SiO_2_@Zn before and after soaking in 2 M ZnSO_4_ for 7 days.

**
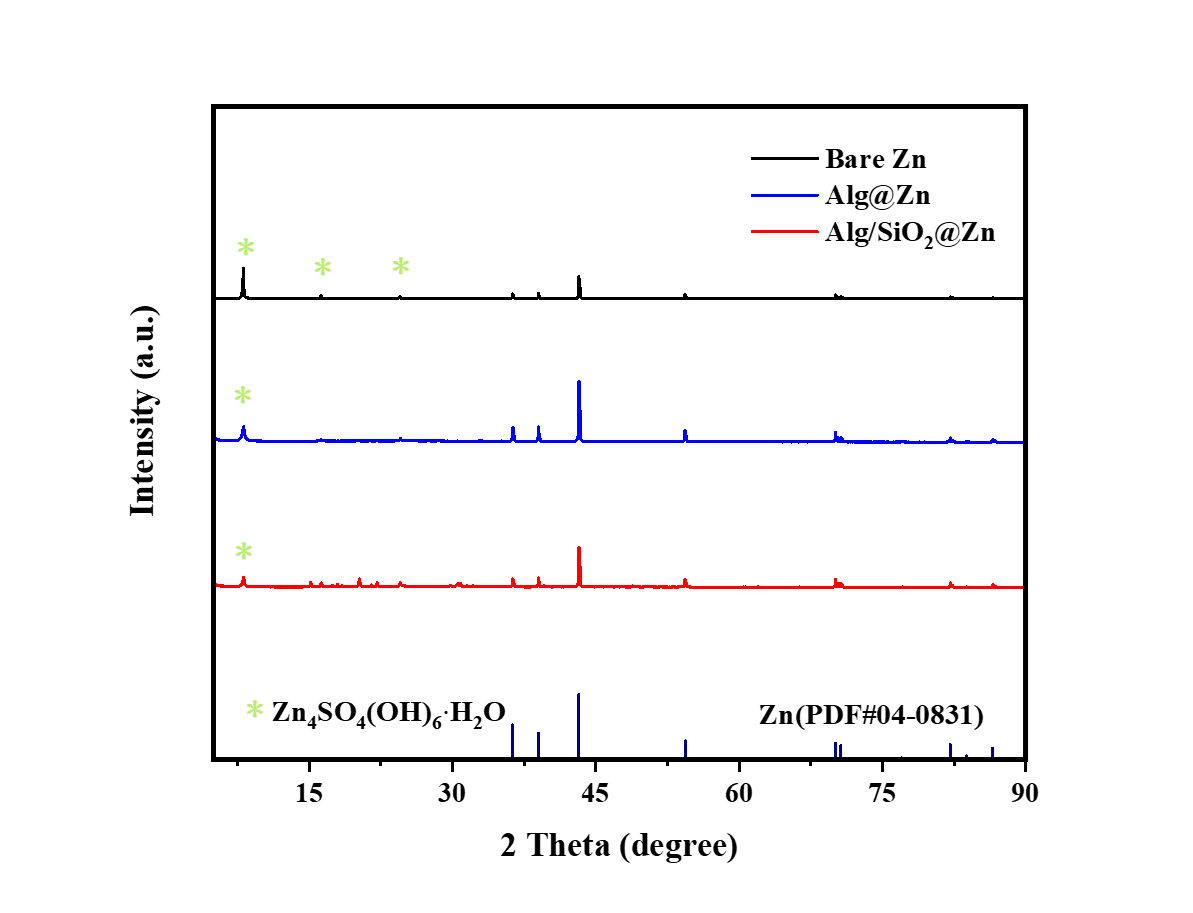
**

**Figure S9.** XRD of bare Zn, Alg@Zn and Alg/SiO_2_@Zn after 7 days of soaking in 2 M ZnSO_4_.

**
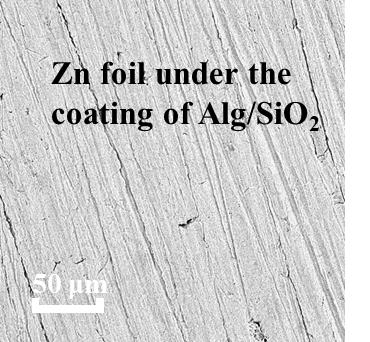
**

**Figure S10.** SEM of Zn surface after removal of coating after 7 days of Alg/SiO_2_@Zn immersion in electrolyte.

**
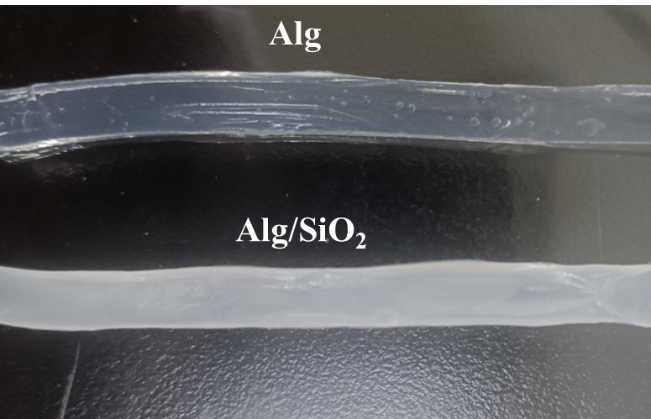
**

**Figure S11** Optical photographs of Alg and Alg/SiO_2_ gels.

**
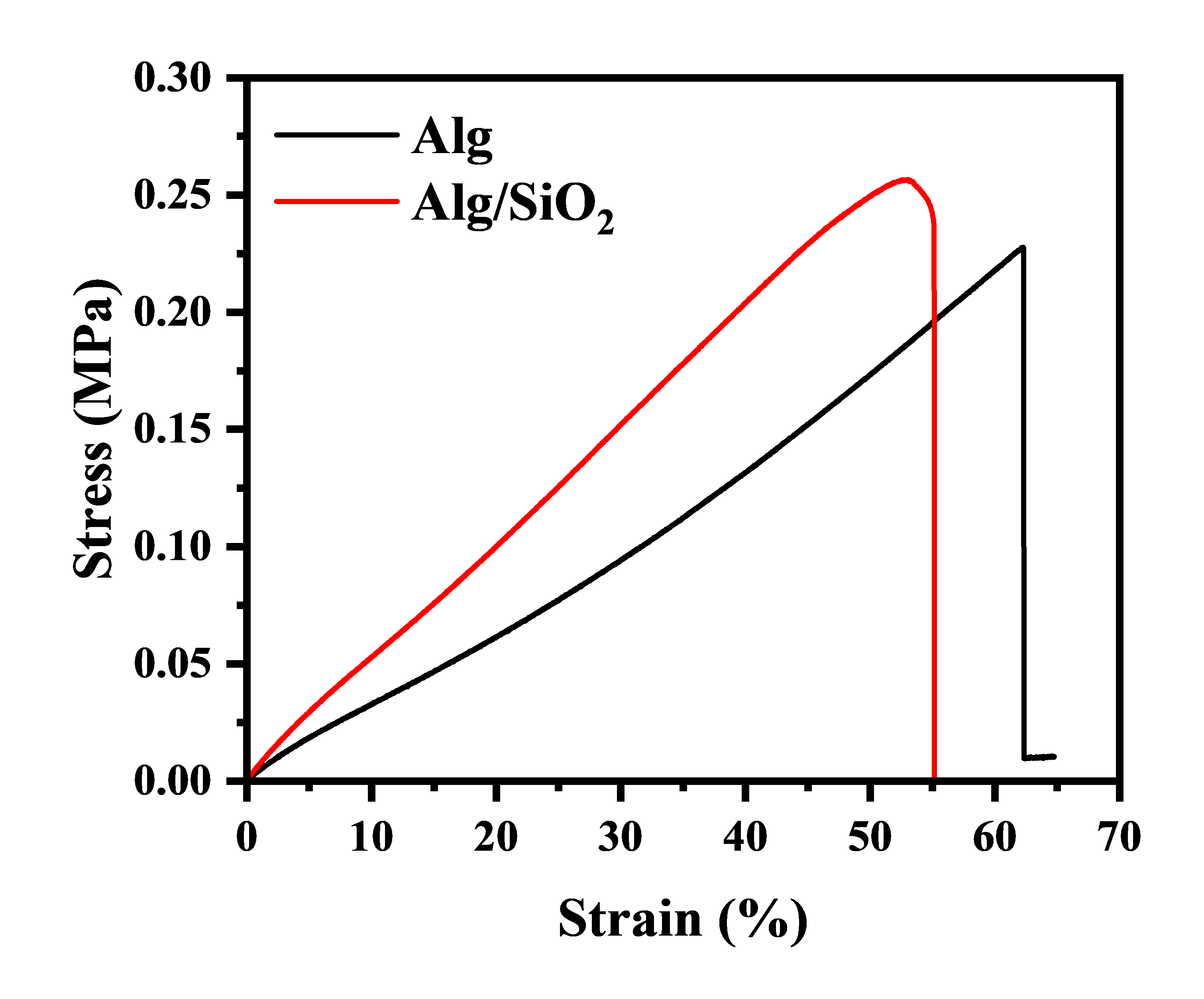
**

**Figure S12.** Stress-strain curves of Alg and Alg/SiO_2_ gels.


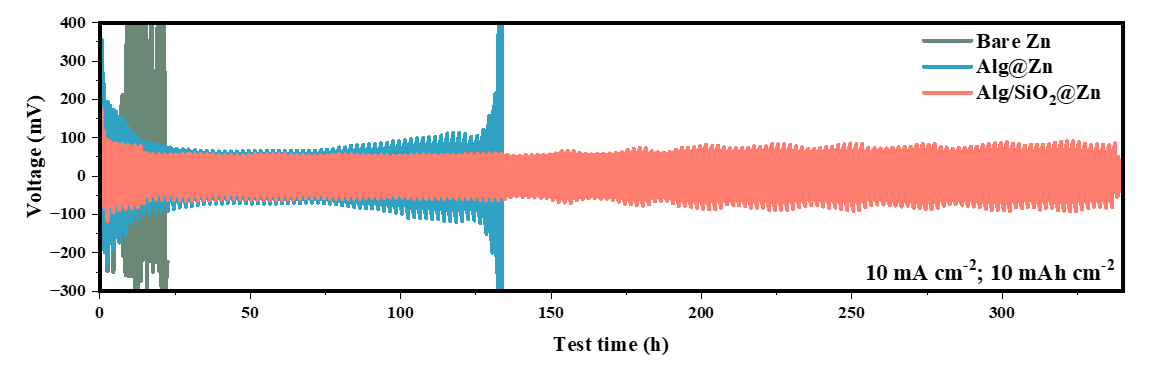


**Figure S13.** Galvanostatic charge–discharge profiles of different Zn electrodes at a current density of 10 mA cm^-2^ and areal capacity of 10 mAh cm^-2^.


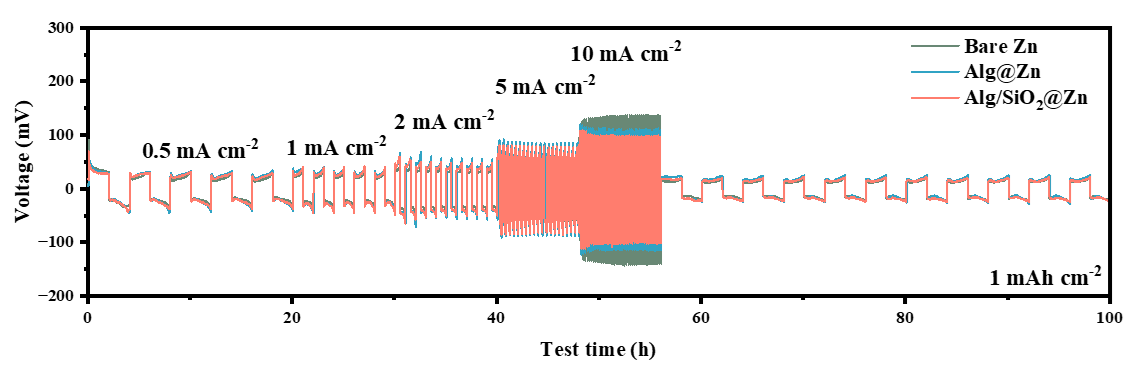


**Figure S14.** Galvanostatic charge–discharge profiles of different Zn electrodes at different current densities.





**Figure S15.** Galvanostatic charge–discharge profile of PVDF/SiO_2_@Zn at a current density of 1 mA cm^-2^ and areal capacity of 1 mAh cm^-2^.





**Figure S16.** Galvanostatic charge–discharge profiles of PVDF/SiO_2_@Zn at different current densities.


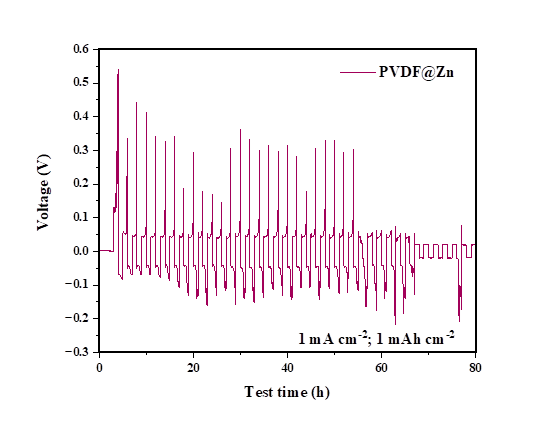


**Figure S17.** Galvanostatic charge–discharge profile of PVDF@Zn at a current density of 1 mA cm^-2^ and areal capacity of 1 mAh cm^-2^.


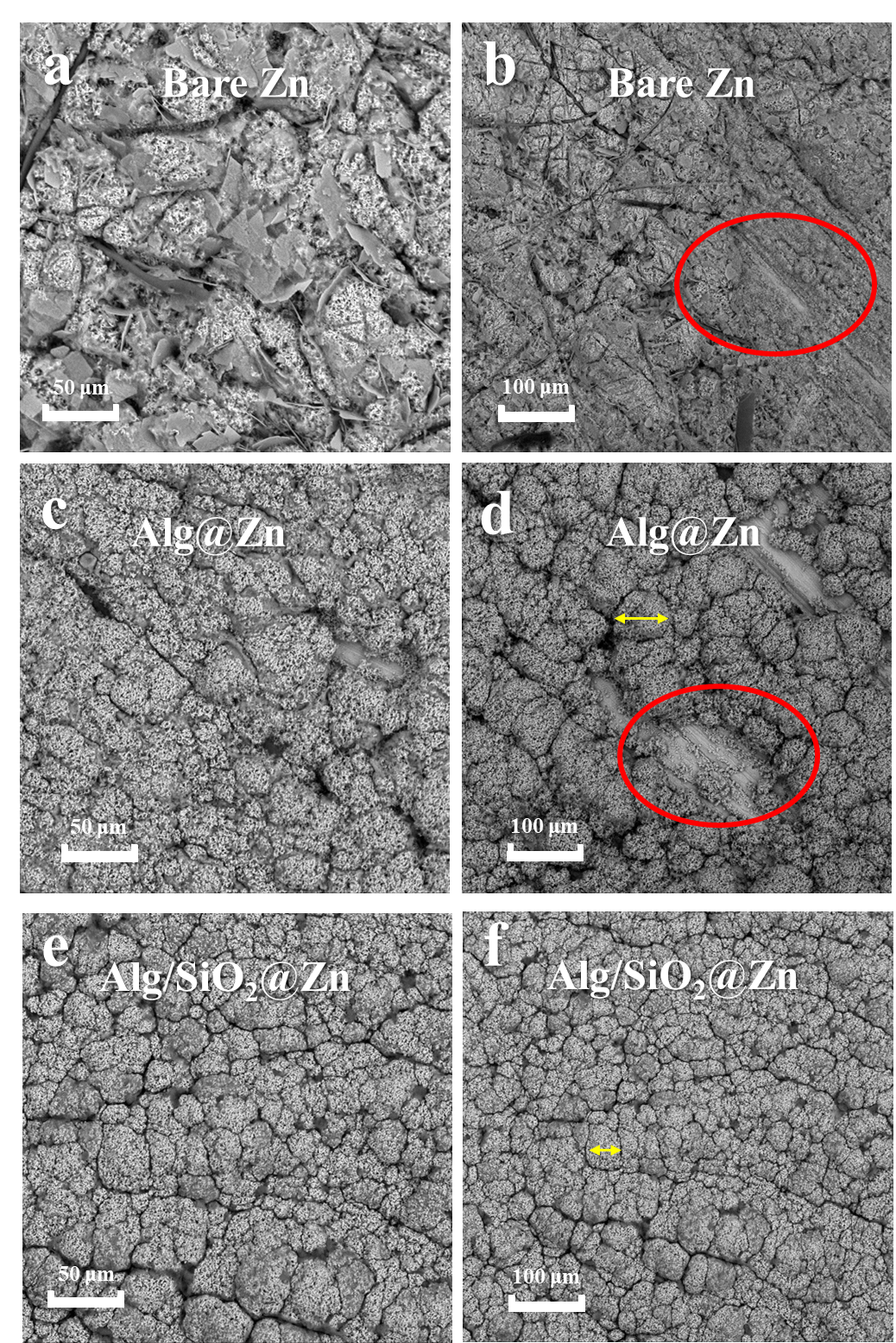


**Figure S18.** SEM images of (a, b) bare Zn, (c, d) Alg@Zn and (e, f) Alg/SiO_2_@Zn after plating for 10 h at 1 mA cm^-2^ current density and removal of coating. (The red circle shows areas where no zinc has been deposited and the yellow line segments represent individual nucleation diameters.)


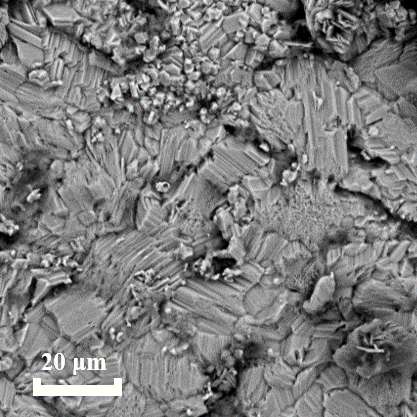


**Figure S19.** SEM of Zn foil with Alg coating removed after 50 cycles (100 h).

**
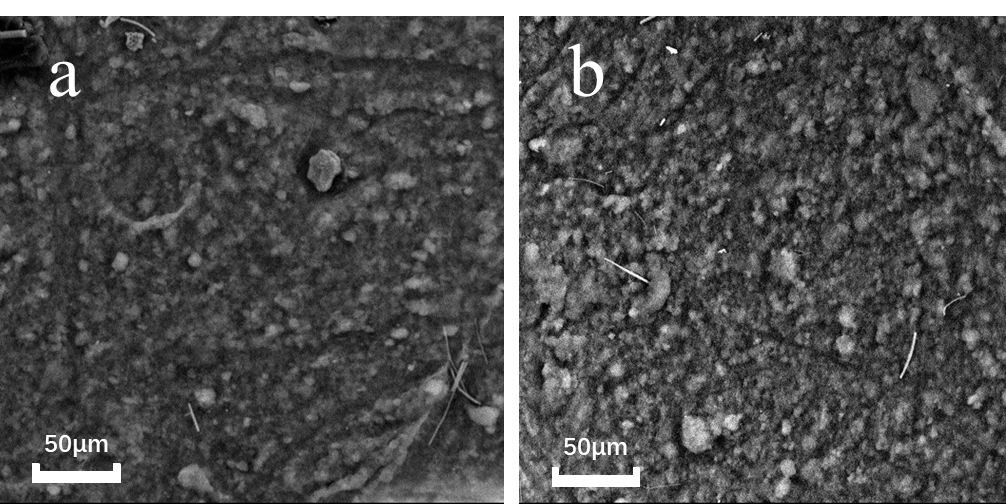
**

**Figure S20.** SEM image of Alg/SiO_2_ coating before (a) and after (b) 50 cycles (100 h) at 1 mA cm^-2^ current density and 1 mAh cm^-2^ areal capacity.

**
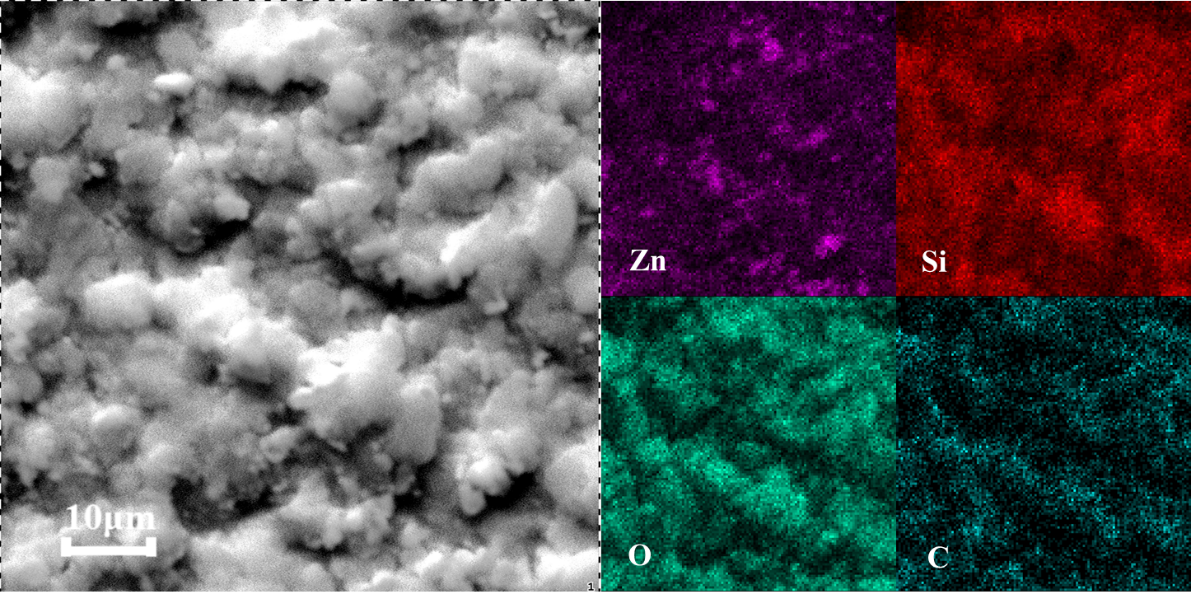
**

**Figure S21.** EDS of Alg/SiO_2_ coating after 50 cycles (100 h).


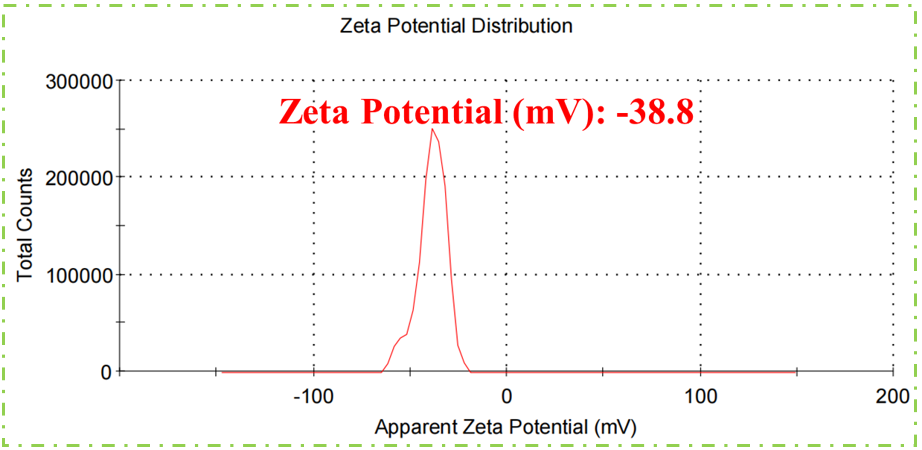


**Figure S22.** Zeta potential of SiO_2_ dispersed in deionised water.

**In situ deposition characterization**

A visual approach was used to further demonstrate the inhibitory effect of Alg/SiO_2_ coatings on zinc dendrites compared to bare Zn. In situ optical microscopy was used to characterize the deposition process of Zn ions to investigate the deposition behavior of the different Zn anode. At a current density of 0.3 mA cm^-2^, bare Zn already exhibits inhomogeneous deposits on its surface after 15 minutes, and a large number of irregular morphologies is present after 30 minutes deposition. Spherical bumps are also clearly observed for Alg@Zn after 30 minutes deposition, which is not beneficial for long-term cycling. In contrast, Alg/SiO_2_@Zn exhibits a flat surface during the whole deposition process, suggesting that Alg/SiO_2_ coating is efficient in guiding the uniform Zn deposition (Figure S23).


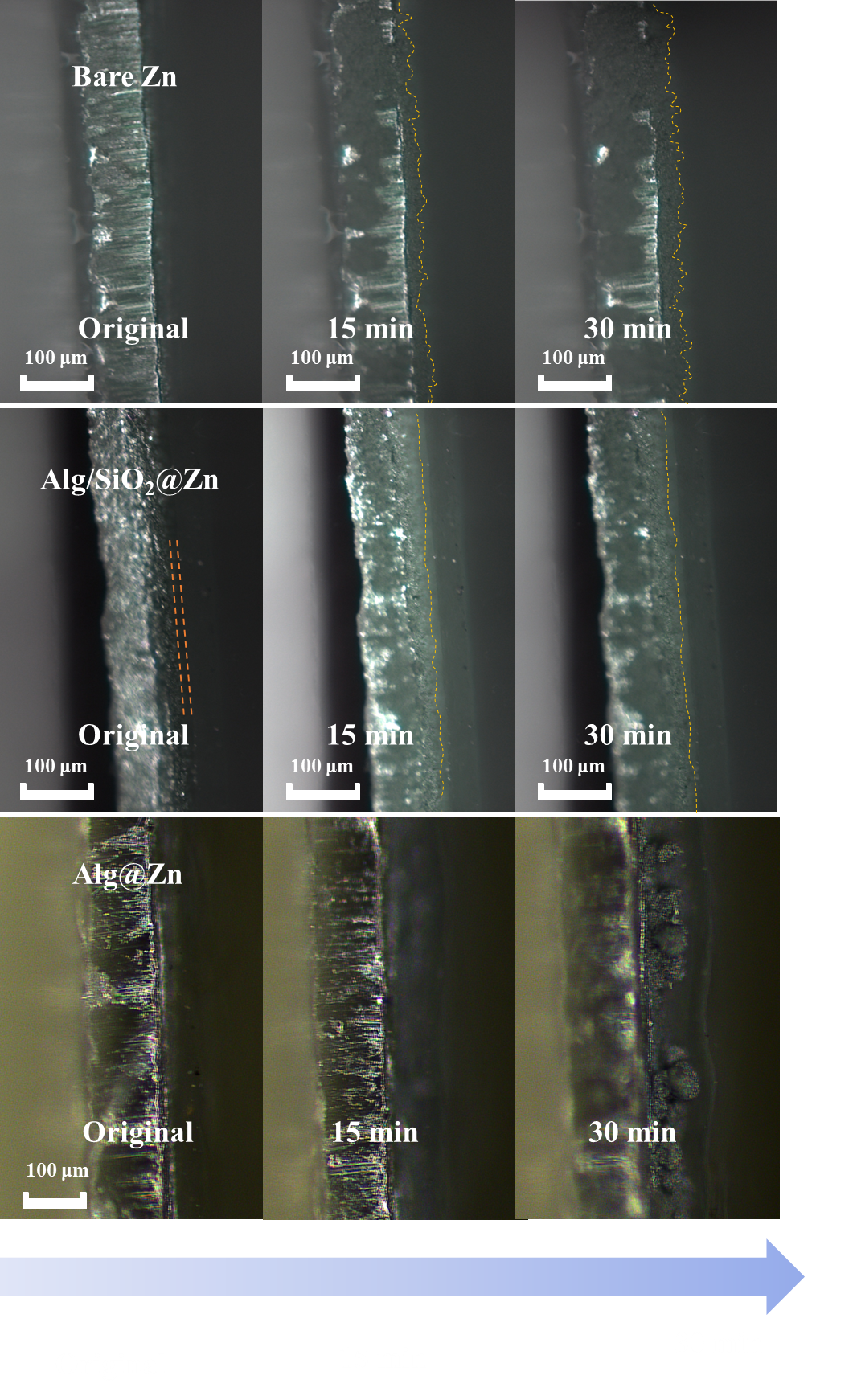


**Figure S23.** In situ optical observation results of the Zn deposition morphologies on different Zn anodes at 0.3 mA cm^-2^.


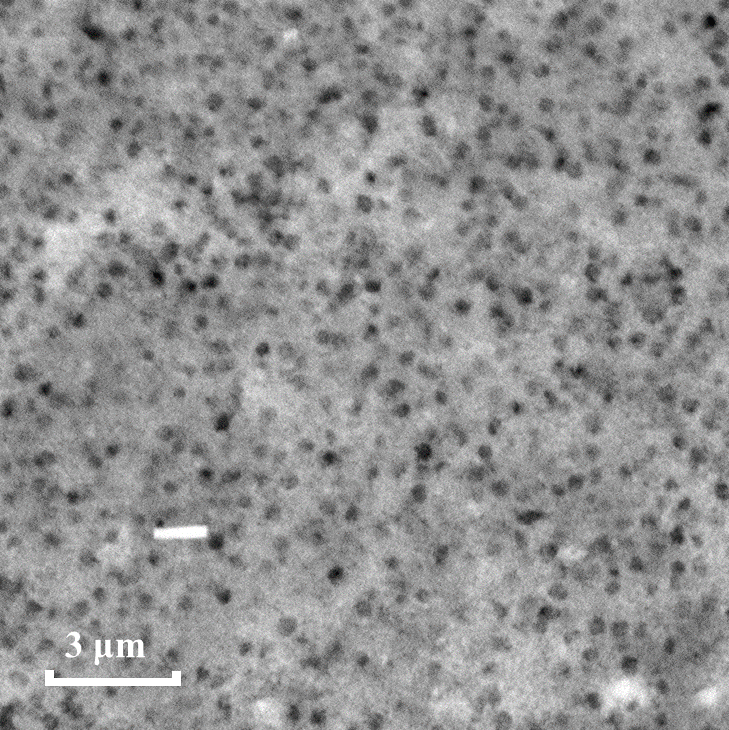


**Figure S24.** SEM of PVDF/SiO_2_ coating after 50 cycles (100 h).


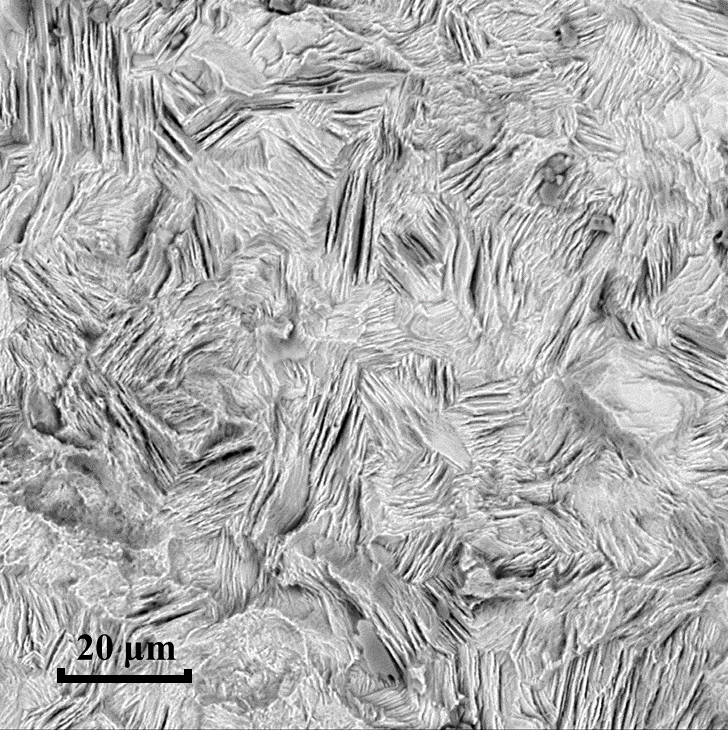


**Figure S25.** SEM of Zn foil with PVDF/SiO_2_ coating removed

after 50 cycles (100 h).


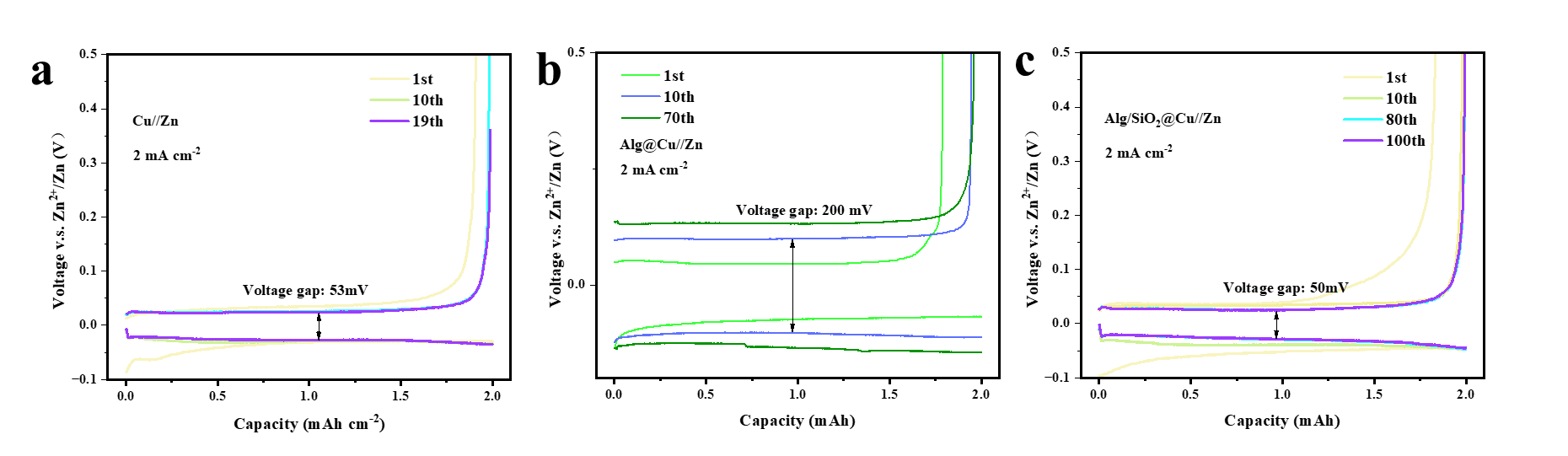


**Figure S26** The typical GCD profiles of (a) Cu//Zn, (b) Alg@Cu//Zn and (c) Alg/SiO_2_@Cu//Zn asymmetric cells at a current density of 2 mA cm^-2^ and an area capacity of 2 mAh cm^-2^.


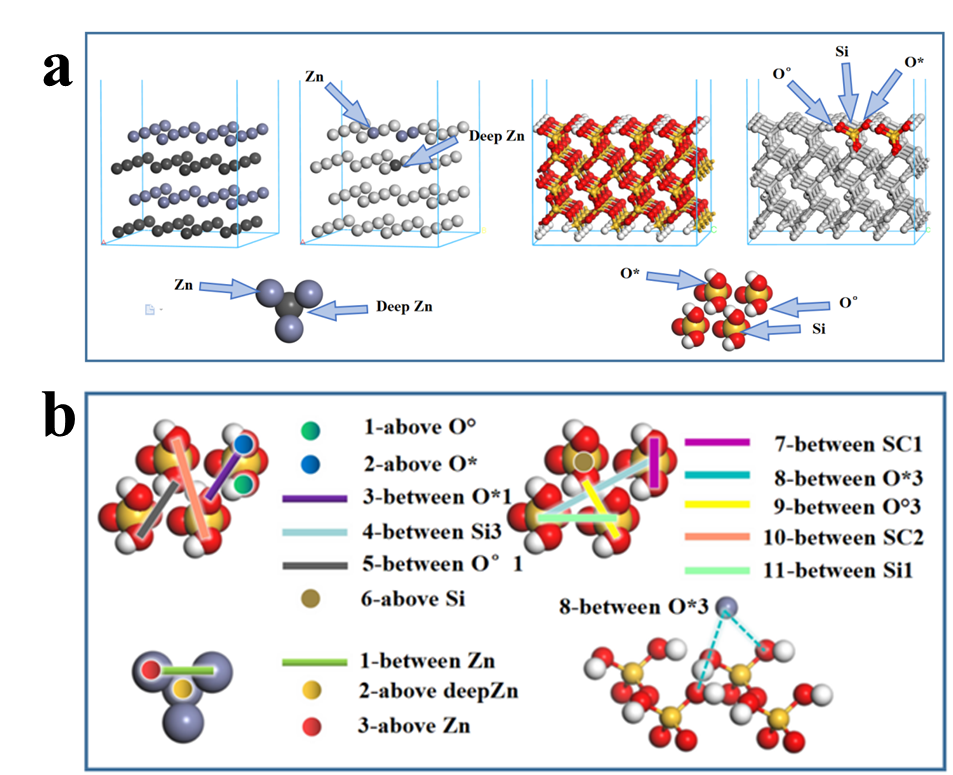


**Figure S27.** (a) Lattice structure of Zn (002) surface (left) and OH-passivated SiO_2_ (001) surface (right). To accurately indicate all the different sites, the established Zn (002) crystal plane contains two layers of zinc atoms. The Zn atoms (surface) and the Deep Zn atoms are labeled and presented as grey and black balls, respectively. The Si atoms, the oxygen and the Hydrogen atoms are presented as yellow, red and white balls. (b) Special site selection on Zn (002) and SiO_2_ (001) surfaces. The Si atoms, zinc atoms the oxygen and the Hydrogen atoms are presented as yellow, grey, red and white balls. The dot represents above the atom and the line represents between the two atoms.


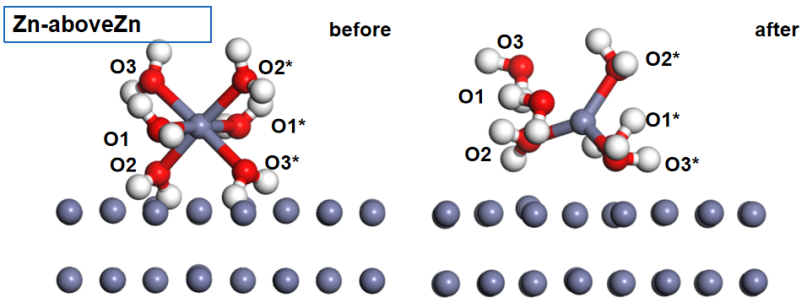


**Figure S28a.** Initial (left) and optimized (right) geometry structure of [Zn(H_2_O)_6_]^2+^ structure at the above-Zn site.


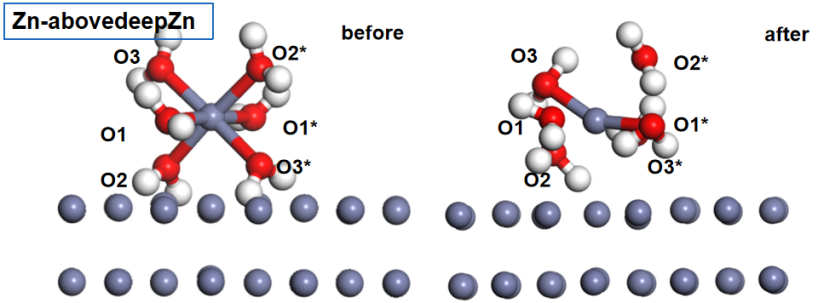


**Figure S28b.** Initial (left) and optimized (right) geometry structure of [Zn(H_2_O)_6_]^2+^ structure at the above-deep-Zn site.


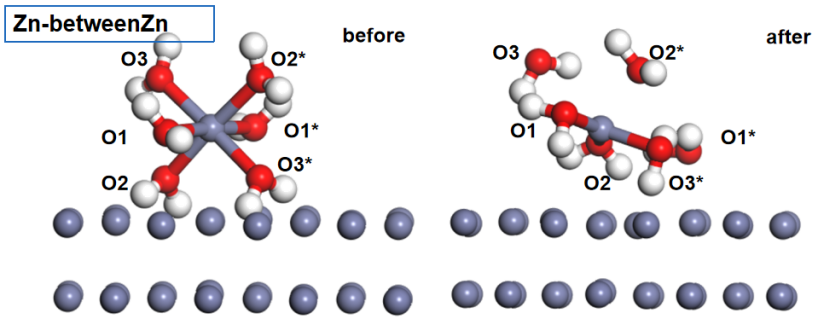


**Figure S28c.** Initial (left) and optimized (right) geometry structure of [Zn(H_2_O)_6_]^2+^ structure at the between-Zn site.


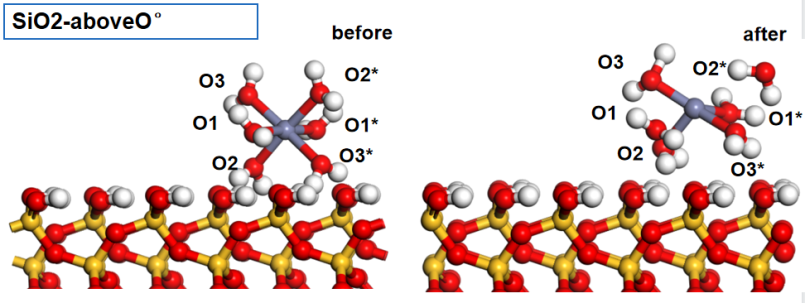


**Figure S28d.** Initial (left) and optimized (right) geometry structure of [Zn(H_2_O)_6_]^2+^ structure at the above-O° site.


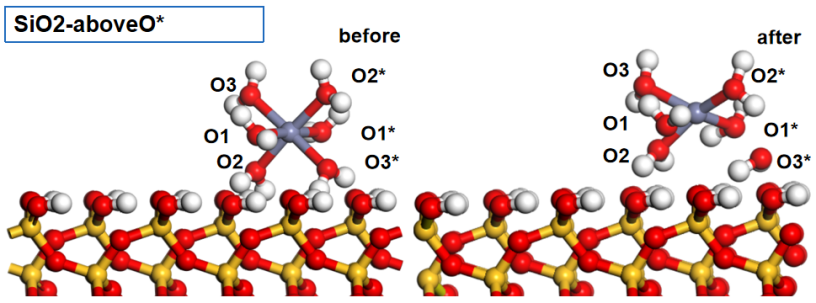


**Figure S28e.** Initial (left) and optimized (right) geometry structure of [Zn(H_2_O)_6_]^2+^ structure at the above-O* site.


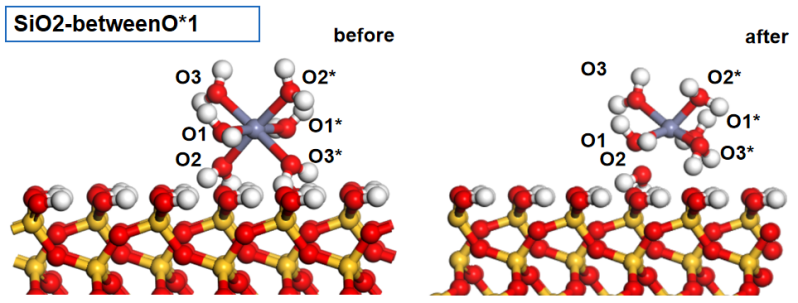


**Figure S28f.** Initial (left) and optimized (right) geometry structure of [Zn(H_2_O)_6_]^2+^ structure at the between-O*1 site.


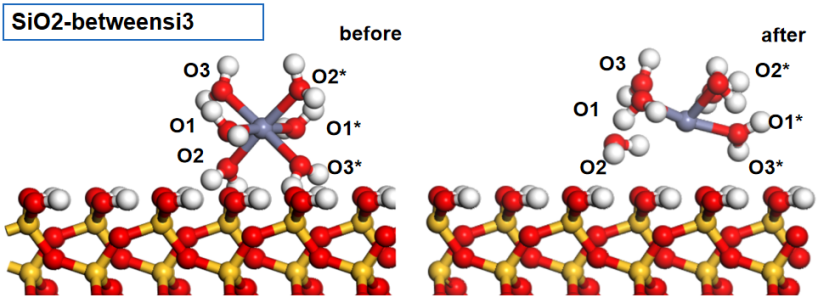


**Figure S28g.** Initial (left) and optimized (right) geometry structure of [Zn(H_2_O)_6_]^2+^ structure at the between-Si3 site.


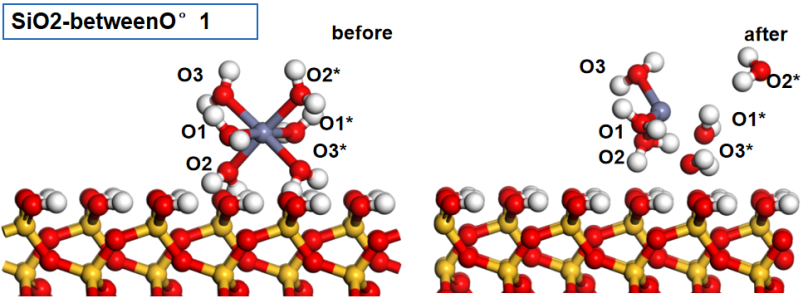


**Figure S28h.** Initial (left) and optimized (right) geometry structure of [Zn(H_2_O)_6_]^2+^ structure at the between- O° 1 site.


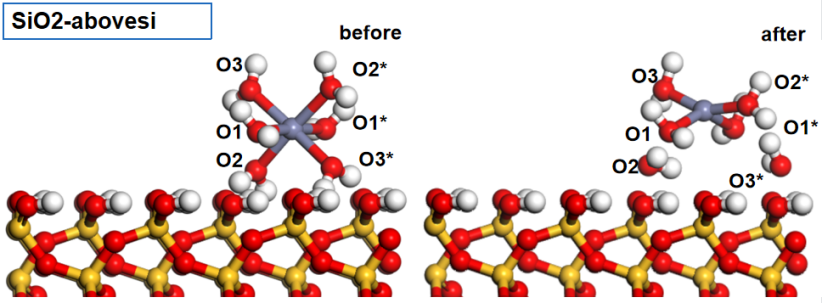


**Figure S28i.** Initial (left) and optimized (right) geometry structure of [Zn(H_2_O)_6_]^2+^ structure at the above-Si site.


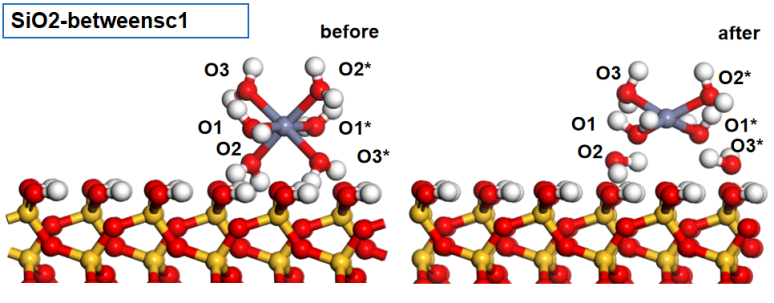


**Figure S28j.** Initial (left) and optimized (right) geometry structure of [Zn(H_2_O)_6_]^2+^ structure at the between-SC1 site.


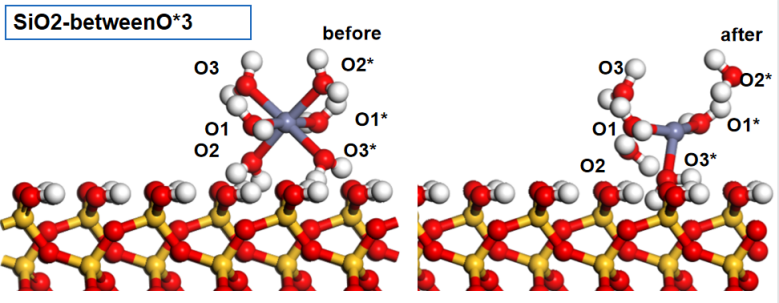


**Figure S28k.** Initial (left) and optimized (right) geometry structure of [Zn(H_2_O)_6_]^2+^ structure at the between-O*3 site.


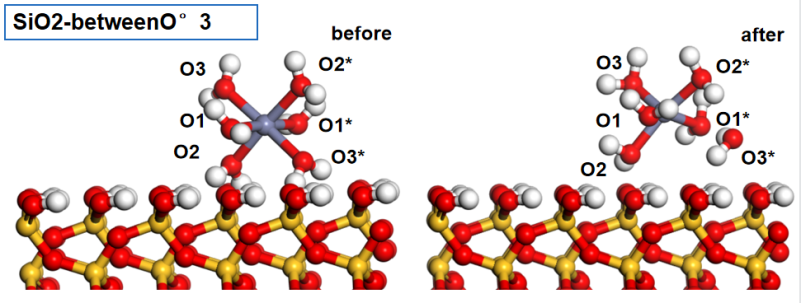


**Figure S28l.** Initial (left) and optimized (right) geometry structure of [Zn(H_2_O)_6_]^2+^ structure at the between-O° 3 site.


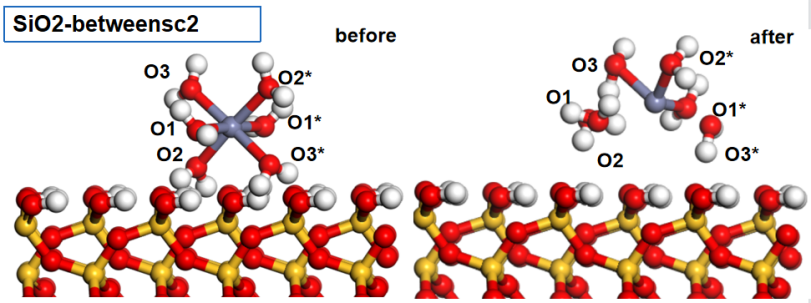


**Figure S28m.** Initial (left) and optimized (right) geometry structure of [Zn(H_2_O)_6_]^2+^ structure at the between-SC2 site.


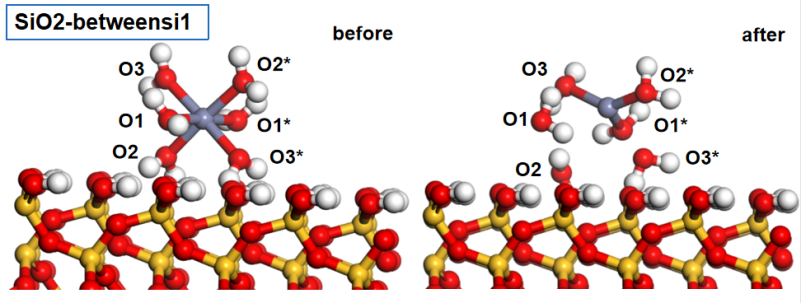


**Figure S28n.** Initial (left) and optimized (right) geometry structure of [Zn(H_2_O)_6_]^2+^ structure at the between-Si1 site.


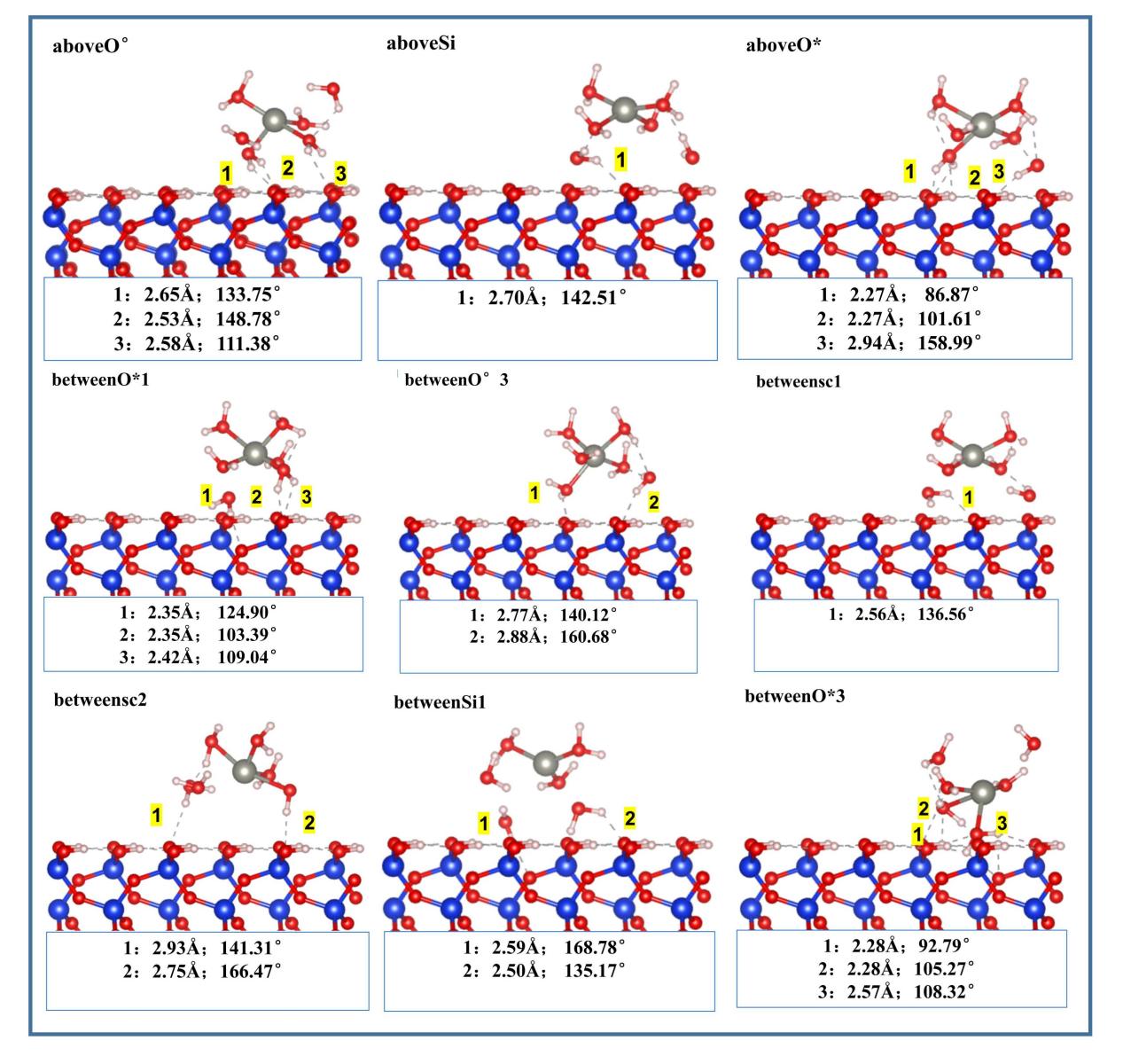


**Figure S29.** H-bonds between H_2_O molecules in the water sheath and the O-H chains on the SiO_2_ (001) surface. Yellow markings indicate the location of hydrogen bonds. The parameters of the measured hydrogen bond are given in the box. The Si atoms, the Oxygen, the Zinc and the Hydrogen atoms are presented as blue, red, grey and white balls.


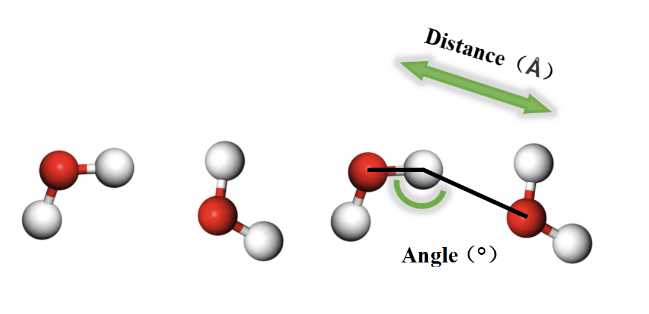


**Figure S30.** Hydrogen atoms are presented as red and white balls.

The definition of hydrogen bonding typically involves two measures: the O-O distance between two water molecules (approximately 3Å) and the angle of O-H-O (around 150°), as demonstrated. These two criteria are commonly used to determine whether a bond formed is a hydrogen bond.^[31]^


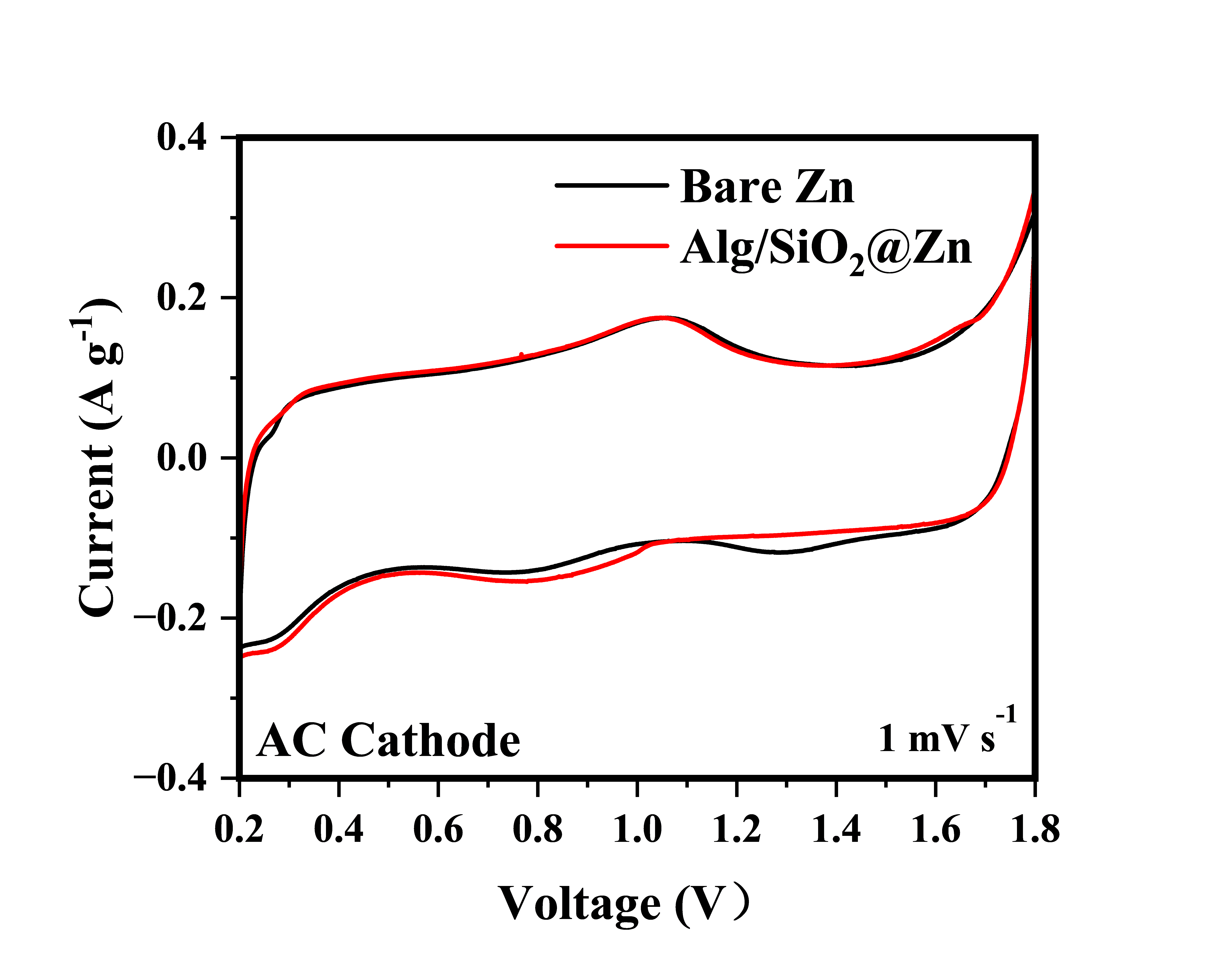


**Figure S31.** CV of AC in the Zn//AC full cell with different Zn anodes in the voltage range of 0.2-1.8 V.


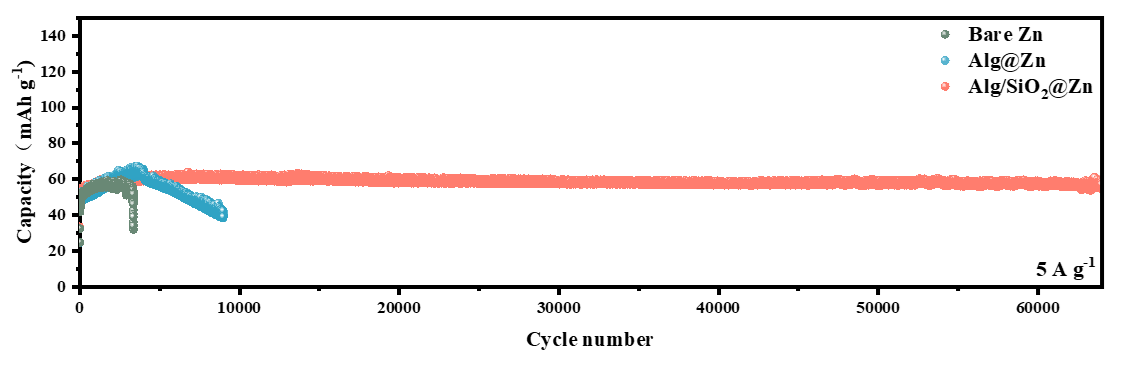


**Figure S32.** Long-term cycling stability of bare Zn//AC, Alg@Zn//AC and Alg/SiO_2_@Zn//AC full cells at 5 A g^−1^.


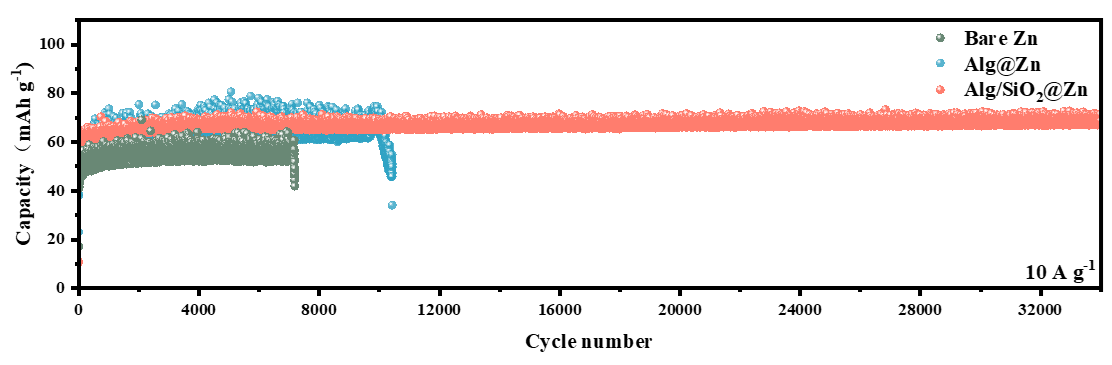


**Figure S33.** Long-term cycling stability of bare Zn//AC, Alg@Zn//AC and Alg/SiO_2_@Zn//AC full cells at 10 A g^−1^.


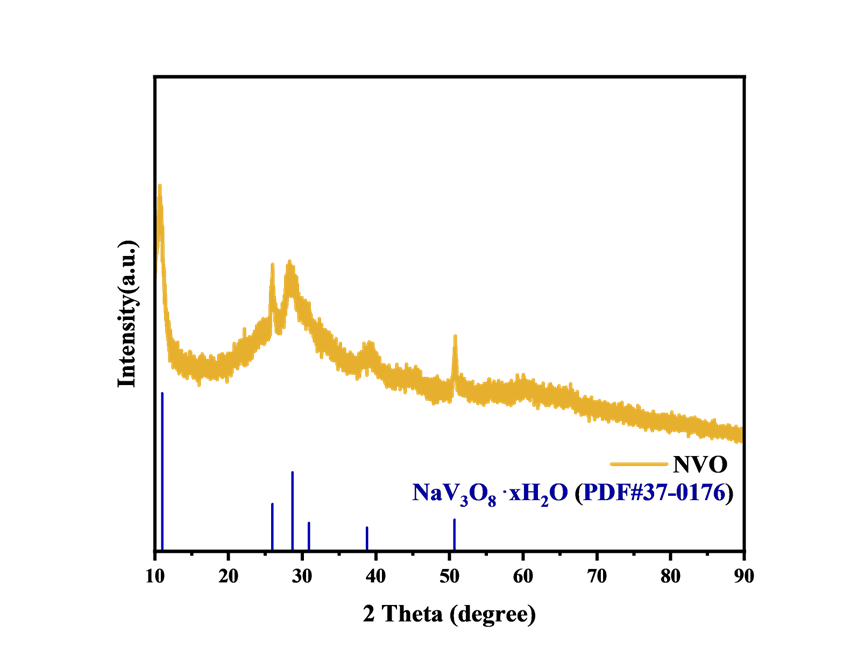


**Figure S34.** XRD of nanowire-type NaV_3_O_8_·*x*H_2_O cathode material.


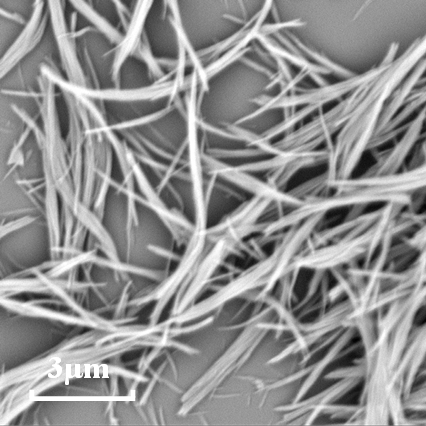


**Figure S35.** SEM of nanowire-type NaV_3_O_8_·*x*H_2_O cathode material.


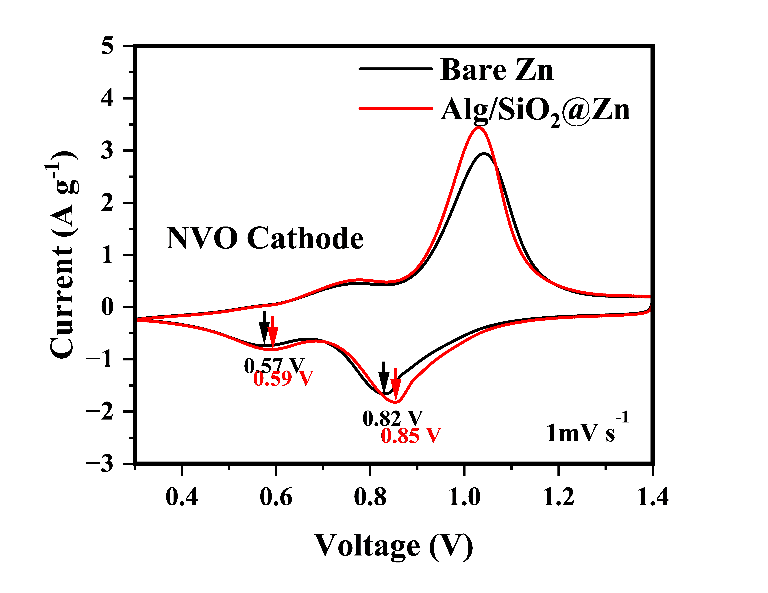


**Figure S36.** CV of NVO in the Zn//NVO full cell with different Zn anodes in the voltage range of 0.3-1.4 V.


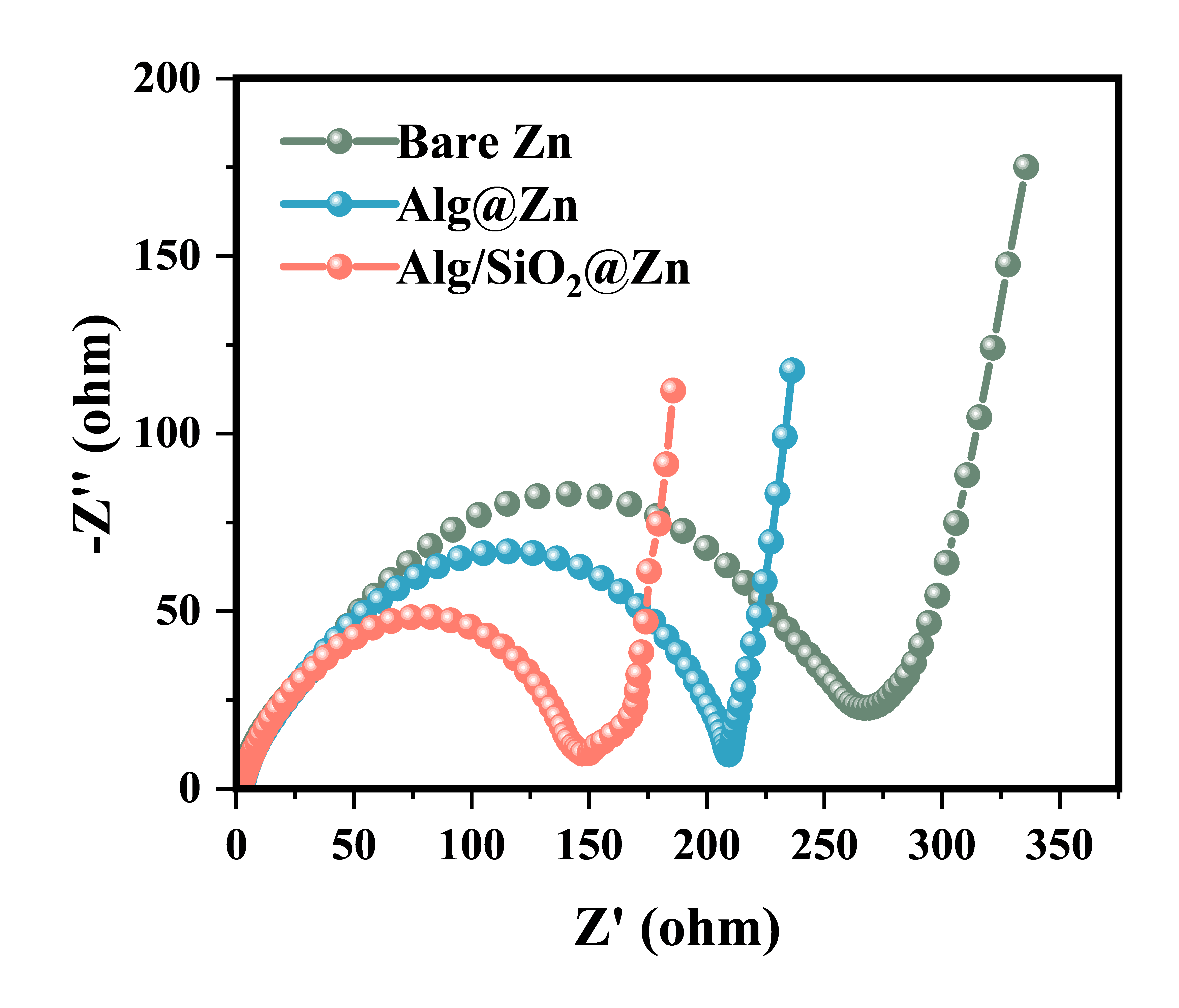


**Figure S37.** EIS for bare Zn//NVO, Alg@Zn//NVO and Alg/SiO_2_@Zn//NVO before cycling.


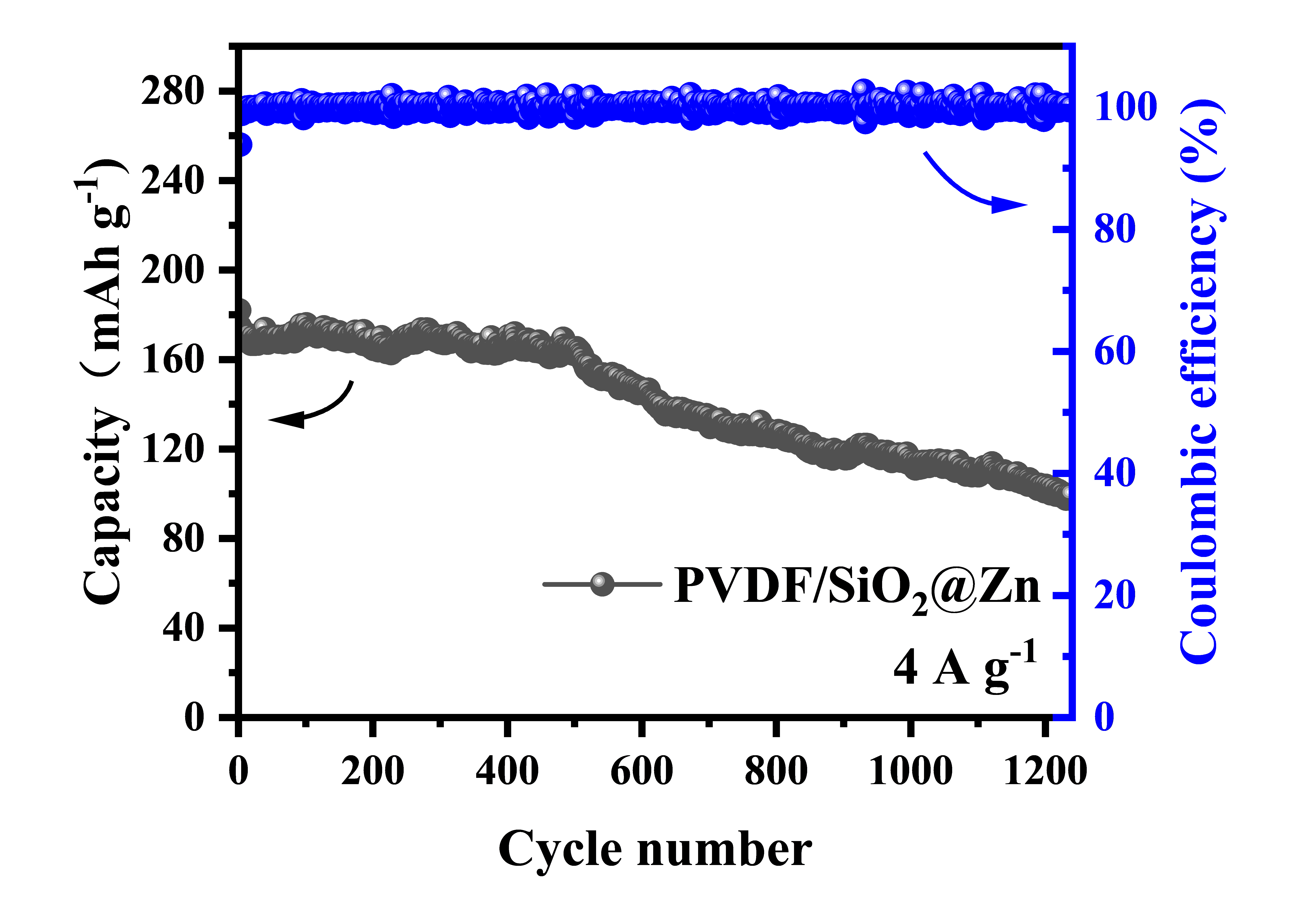


**Figure S38.** Long-term cycling stability of PVDF/SiO_2_@Zn//NVO

full cell at 4 A g^−1^.


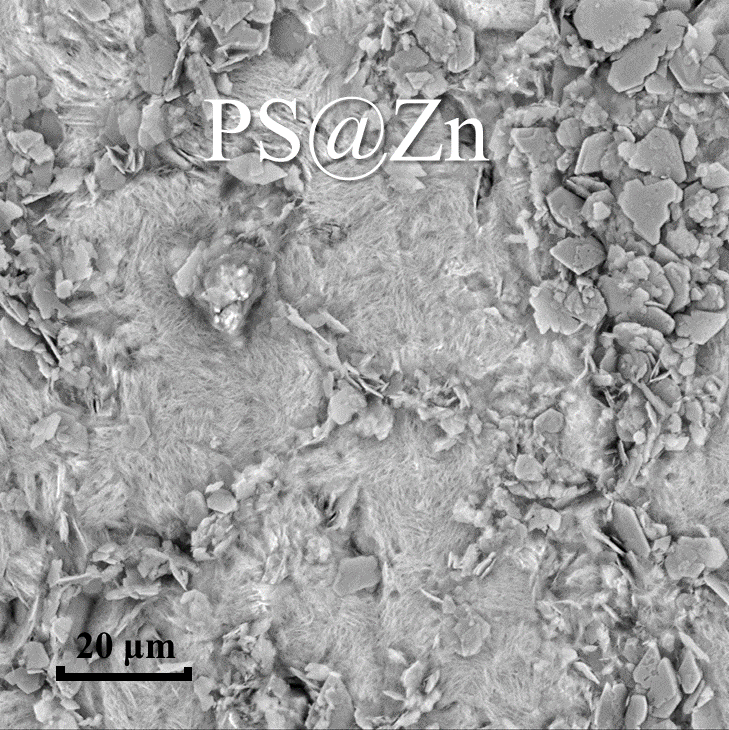


**Figure S39.** SEM of PVDF/SiO_2_@Zn anode after 200 cycles of the full cell with NVO as cathode.

REFERENCES

[1] T. D. Kühne, M. Iannuzzi, M. Del Ben, V. V. Rybkin, P. Seewald, F. Stein, T. Laino, R. Z. Khaliullin, O. Schütt, F. Schiffmann, D. Golze, J. Wilhelm, S. Chulkov, M. H. Bani-Hashemian, V. Weber, U. Borštnik, M. Taillefumier, A. S. Jakobovits, A. Lazzaro, H. Pabst, T. Müller, R. Schade, M. Guidon, S. Andermatt, N. Holmberg, G. K. Schenter, A. Hehn, A. Bussy, F. Belleflamme, G. Tabacchi, A. Glöß, M. Lass, I. Bethune, C. J. Mundy, C. Plessl, M. Watkins, J. VandeVondele, M. Krack, J. Hutter, *J. Chem. Phys.* **2020**, *152*, 194103.

[2] J. VandeVondele, J. Hutter, *J. Chem. Phys.* **2007**, *127*, 114105.

[3] a) C. Hartwigsen, S. Goedecker, J. Hutter, *PHYS. REV. B* **1998**, *58*, 3641-3662; b) S. Grimme, J. Antony, S. Ehrlich, H. Krieg, *J. Chem. Phys.* **2010**, *132*, 154104.

[4] Q. Zhang, J. Luan, X. Huang, Q. Wang, D. Sun, Y. Tang, X. Ji, H. Wang, *Nat. Commun.* **2020**, *11*, 3961.

[5] K. Zhao, C. Wang, Y. Yu, M. Yan, Q. Wei, P. He, Y. Dong, Z. Zhang, X. Wang, L. Mai, *Adv. Mater. Interfaces* **2018**, *5*, 1800848.

[6] K. Hu, X. Guan, R. Lv, G. Li, Z. Hu, L. Ren, A. Wang, X. Liu, J. Luo, *Chem. Eng. J.* **2020**, *396*, 125363.

[7] M. Liu, J. Cai, H. Ao, Z. Hou, Y. Zhu, Y. Qian, *Adv. Funct. Mater.* **2020**, *30*, 2004885.

[8] J. Y. Kim, G. Liu, G. Y. Shim, H. Kim, J. K. Lee, *Adv. Funct. Mater.* **2020**, *30*, 2004210.

[9] L. Dai, T. Wang, B. Jin, N. Liu, Y. Niu, W. Meng, Z. Gao, X. Wu, L. Wang, Z. He, *Surf. Coat. Technol.* **2021**, *427*, 127813.

[10] Q. Lu, C. Liu, Y. Du, X. Wang, L. Ding, A. Omar, D. Mikhailova, *ACS Appl. Mater. Interfaces* **2021**, *13*, 16869-16875.

[11] M. Zhou, S. Guo, G. Fang, H. Sun, X. Cao, J. Zhou, A. Pan, S. Liang, *J. Energy Chem.* **2021**, *55*, 549-556.

[12] Y. Yang, C. Liu, Z. Lv, H. Yang, Y. Zhang, M. Ye, L. Chen, J. Zhao, C. C. Li, *Adv. Mater.* **2021**, *33*, 2007388.

[13] L. Ma, Q. Li, Y. Ying, F. Ma, S. Chen, Y. Li, H. Huang, C. Zhi, *Adv. Mater.* **2021**, *33*, 2007406.

[14] X. Zeng, J. Mao, J. Hao, J. Liu, S. Liu, Z. Wang, Y. Wang, S. Zhang, T. Zheng, J. Liu, P. Rao, Z. Guo, *Adv. Mater.* **2021**, *33*, 200746.

[15] Z. Cao, X. Zhu, D. Xu, P. Dong, M. O. L. Chee, X. Li, K. Zhu, M. Ye, J. Shen, *Energy Storage Mater.* **2021**, *36*, 132-138.

[16] Y. Cao, W. Gu, X. Huang, H. Cao, M. Yang, Y. Long, P. Wu, Y. Yang, Y. Zeng, Y. Luo, L. Zhang, Q. Zheng, D. Lin, *Electrochim. Acta* **2023**, *470*, 143286.

[17] H. He, J. Liu, *J. Mater. Chem. A* **2020**, *8*, 22100-22110.

[18] X. Han, H. Leng, Y. Qi, P. Yang, J. Qiu, B. Zheng, J. Wu, S. Li, F. Huo, *Chem. Eng. J.* **2022**, *431*, 133931.

[19] Y. Gao, M. Wang, Y. Chu, X. Li, J. Li, J. Chen, Z. Ma, B. Guo, B. Yu, Y. Pan, Y. Huang, G. Cao, X. Li, *Small* **2024**, 2405139.

[20] T. Wang, H. Fu, S. Tang, W. Xiang, M. Li, J. K. Lee, L. Xu, J. S. Yu, *Chem. Eng. J.* **2024**, *493*, 152577.

[21] M. Cui, B. Yan, F. Mo, X. Wang, Y. Huang, J. Fan, C. Zhi, H. Li, *Chem. Eng. J.* **2022**, *434*, 134688.

[22] X. Lei, Z. Ma, L. Bai, L. Wang, Y. Ding, S. Song, A. Song, H. Dong, H. Tian, H. Tian, X. Meng, H. Liu, B. Sun, G. Shao, G. Wang, *Battery Energy* **2023**, *2*, 20230024.

[23] Z. Zhao, J. Zhao, Z. Hu, J. Li, J. Li, Y. Zhang, C. Wang, G. Cui, *Energy Environ. Sci.* **2019**, *12*, 1938-1949.

[24] A. Chen, C. Zhao, J. Gao, Z. Guo, X. Lu, J. Zhang, Z. Liu, M. Wang, N. Liu, L. Fan, Y. Zhang, N. Zhang, *Energy Environ. Sci.* **2023**, *16*, 275-284.

[25] W. Guo, Z. Cong, Z. Guo, C. Chang, X. Liang, Y. Liu, W. Hu, X. Pu, *Energy Storage Mater.* **2020**, *30*, 104-112.

[26] W. Deng, Z. Xu, X. Wang, *Energy Storage Mater.* **2022**, *52*, 52-60.

[27] Y. Yang, S. Guo, Y. Pan, B. Lu, S. Liang, J. Zhou, *Energy Environ. Sci.* **2023**, *16*, 2358-2367.

[28] B. Qiu, L. Xie, G. Zhang, K. Cheng, Z. Lin, W. Liu, C. He, P. Zhang, H. Mi, *Chem. Eng. J.* **2022**, *449*, 137843.

[29] T. Wei, Y. Ren, Z. Li, X. Zhang, D. Ji, L. Hu, *Chem. Eng. J.* **2022**, *434*, 134646.

[30] X. Cheng, Y. Zuo, Y. Zhang, X. Zhao, L. Jia, J. Zhang, X. Li, Z. Wu, J. Wang, H. Lin, *Adv. Sci.* **2024**, *11*, 2401629.

[31] a) R. Ferreira de Freitas, M. Schapira, *MedChemComm* **2017**, *8*, 1970-1981; b) C. Bissantz, B. Kuhn, M. Stahl, *J. Med. Chem.* **2010**, *53*, 5061-5084.
